# Regioselective electrosynthesis of tetra- and hexa-functionalized [60]fullerene derivatives with unprecedented addition patterns†
†Electronic supplementary information (ESI) available: Detailed experimental procedures, characterization data, calculation results, NMR, UV-vis and fluorescence spectra, CVs and DPVs of **2–5** (PDF). X-ray crystallographic data for **5** (CIF). CCDC 1900924. For ESI and crystallographic data in CIF or other electronic format see DOI: 10.1039/c9sc02131k


**DOI:** 10.1039/c9sc02131k

**Published:** 2019-11-20

**Authors:** Kai-Qing Liu, Jun-Jie Wang, Xing-Xing Yan, Chuang Niu, Guan-Wu Wang

**Affiliations:** a Hefei National Laboratory for Physical Sciences at Microscale , CAS Key Laboratory of Soft Matter Chemistry , iChEM (Collaborative Innovation Center of Chemistry for Energy Materials) , Center for Excellence in Molecular Synthesis of CAS , Department of Chemistry , University of Science and Technology of China , Hefei , Anhui 230026 , P. R. China . Email: gwang@.ustc.edu.cn; b State Key Laboratory of Applied Organic Chemistry , Lanzhou University , Lanzhou , Gansu 730000 , P. R. China

## Abstract

The efficient and regioselective electrosynthesis of tetra- and hexa-functionalized [60]fullerene derivatives with unprecedented addition patterns has been achieved. The tetra-functionalized [60]fullerene derivative with an intriguing 1,2,4,17-addition pattern is regioselectively obtained by cyclization reaction of the dianionic species generated electrochemically from a [60]fulleroindoline with 1,2-bis(bromomethyl)benzene at 0 °C, and can be converted to the more stable 1,2,3,4-adduct at 25 °C. Furthermore, the hexa-functionalized [60]fullerene derivative with the 1,2,3,4,9,10-addition pattern displaying a unique “*S*”-shaped configuration can be synthesized by protonation of the electrochemically generated dianion of the obtained tetra-functionalized 1,2,4,17-adduct. The structures of the tetra- and hexa-functionalized products have been determined by spectroscopic data and single-crystal X-ray analysis.

## Abstract 2

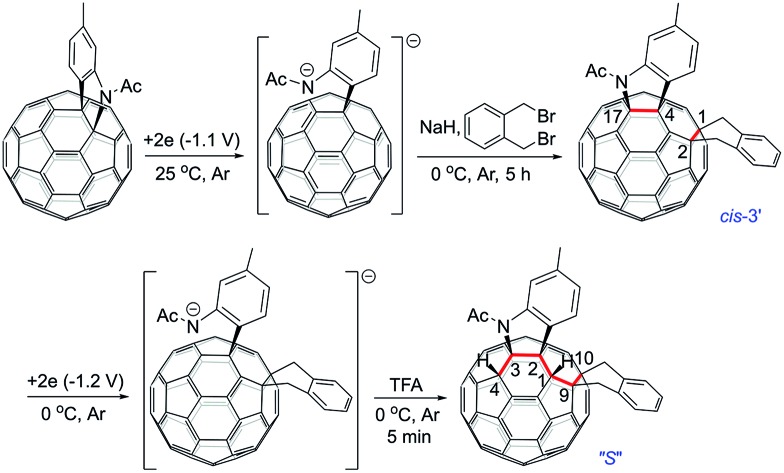
The regioselective electrosynthesis of tetra- and hexa-functionalized [60]fullerene derivatives with unprecedented 1,2,4,17-(*cis*-3′) and 1,2,3,4,9,10-(“*S*”-shaped) addition patterns is achieved.

## Introduction

**Fig. 1:**
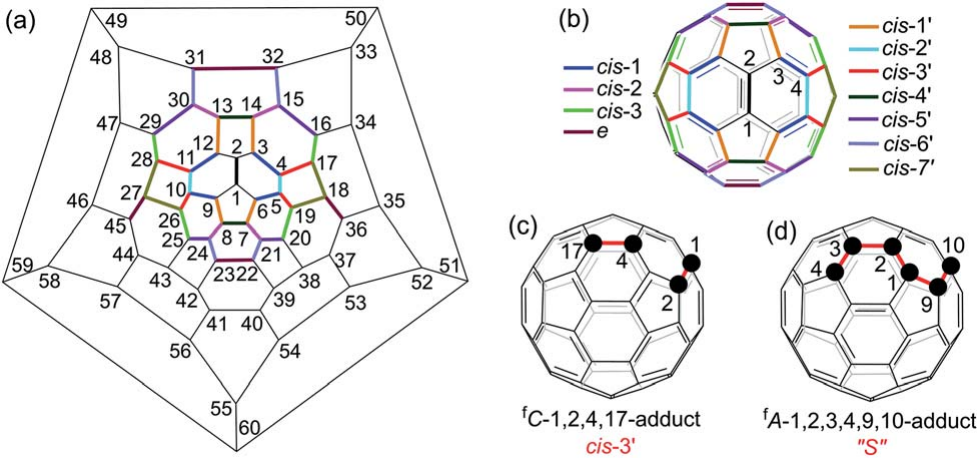
(a) Schlegel diagram of C_60_ with numbering of the C-atoms. (b) The *cis* and *e* isomers fused to two [6,6]-junctions and *cis*′ isomers fixed to a [6,6]-junction and a [5,6]-junction for bis-cycloadducts of C_60_. (c) Structure of the 1,2,4,17-adduct (*cis*-3′). (d) Structure of the “*S*”-shaped 1,2,3,4,9,10-adduct.

Multiply functionalized fullerene derivatives have captured increasing attention for their potential applications in materials and biological science over the past two decades.^1^ Various methods have been established for the synthesis of multi-functionalized [60]fullerene (C_60_) derivatives.^2^ For a [2 + 1]-, [2 + 2]-, [2 + 3]- or [2 + 4]-cycloaddition reaction of C_60_, the cyclization usually gives one monoadduct fused to a [6,6]-junction of C_60_, and affords a mixture of 8 regioisomers for bis-cycloadducts, that is, *cis*-1, *cis*-2, *cis*-3, *e*, *trans*-4, *trans*-3, *trans*-2 and *trans*-1 isomers (Fig. 1b).^3^ Theoretically, there are 46 and 262 possible regioisomers for tris-cycloadducts and tetrakis-cycloadducts, respectively.^2^,^3^ For non-cyclized or partially cyclized tetra-functionalized derivatives of C_60_, the most commonly reported regioisomers are 1,2,3,4-adducts,^4^ 1,4,11,15-adducts,^5^ 1,2,4,15-adducts^6^ and 1,2,3,16-adducts;^7^ meanwhile for the hexa-functionalized derivatives of C_60_, the most frequently reported regioisomers are 1,2,3,4,5,6-adducts^8^ and 1,2,4,11,15,30-adducts^5^,^9^ (Fig. 1a). However, most of the previously reported methods suffer from the problem of poor regioselectivity and difficulty in separation of individual regioisomers. Even though the elegant templated multifunctionalizations of fullerenes have been developed to achieve high regioselectivity for bis-cycloadditions,^3^,^10^ the regioselective formation of a specific isomer of bis-adducts or multi-adducts with new addition patterns is still quite challenging.

Electrosynthesis turns out to be an efficient protocol in organic synthesis due to its mild reaction conditions, good chemoselectivity and regioselectivity, and relatively high yield.^11^,^12^ Furthermore, electrosynthesis has been increasingly applied to the synthesis of multi-functionalized fullerene derivatives including the above-mentioned tetra-functionalized 1,2,3,4-, 1,2,4,15- and 1,2,3,16-adducts in excellent regioselectivity. However, the electrosynthesis has not been applied to the synthesis of hexa-functionalized fullerene derivatives until now. In continuation of our interest in electrochemical functionalizations of C_60_ derivatives,7b,e,f,^13^ particularly the successful synthesis of 1,2,3,16-adducts from the reaction of an electroreduced dianionic [60]fulleroindoline with alkyl bromides7*b* prompted us to explore the reaction of the dianionic species with a bis-electrophile in order to produce cyclized products with novel addition patterns. Herein, we disclose an efficient and straightforward electrosynthesis of tetra-functionalized derivative of C_60_ with an unprecedented 1,2,4,17-addition pattern, also named as *cis*-3′ adduct (Fig. 1b and c),^14^ by the reaction of the dianionic [60]fulleroindoline with 1,2-bis(bromomethyl)benzene. A unique “*S*”-shaped hexa-functionalized derivative with the 1,2,3,4,9,10-addition pattern (Fig. 1d) can be obtained by further protonation of the electrochemically generated dianion of the synthesized 1,2,4,17-adduct.

## Results and discussion

**Fig. 2:**
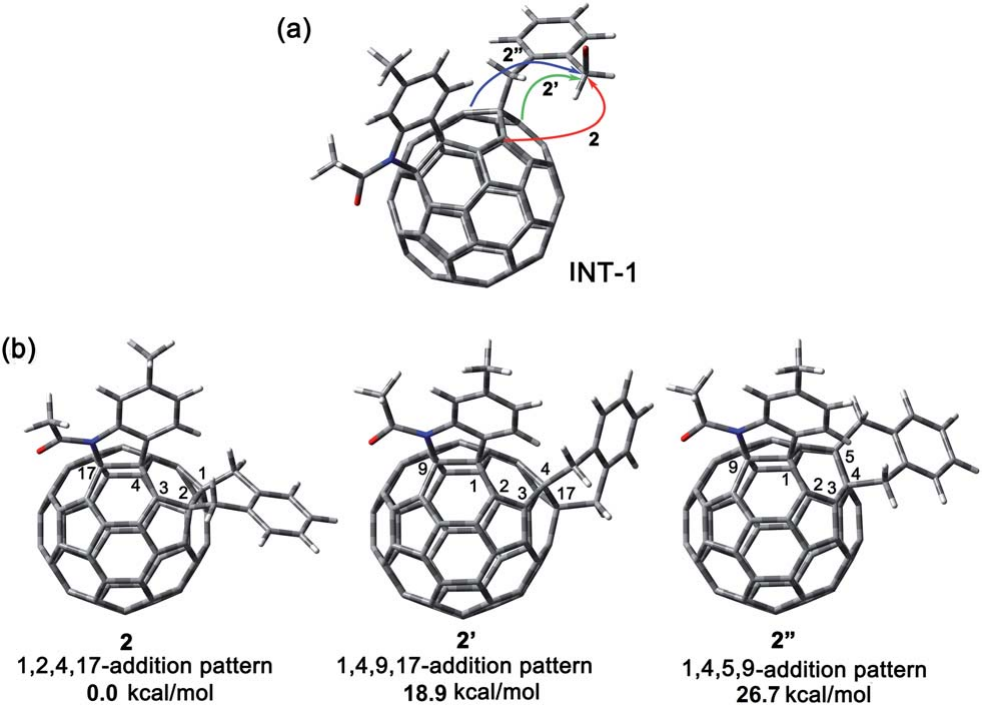
(a) Possible ring-closure pathways of the monoanionic **INT-1**. (b) Relative energies of the possible three ring-closure products **2**, **2′** and **2′′**.

A heterolytic cleavage of the C_60_–N bond would occur after [60]fulleroindoline **1** accepts two electrons, and the dianionic **1^2–^** has its most electronegative carbon atom at the *para* position of the carbon linked with the aryl group, which is more prone to react with an electrophile.7b,e,f We surmise that when **1^2–^** is allowed to react with a bis-electrophile such as 1,2-bis(bromomethyl)benzene, the most electronegative *para* carbon on the fullerene skeleton is expected to attack one of the two –CH_2_Br groups of 1,2-bis(bromomethyl)benzene, followed by a ring-closure process *via* the C_60_–N bond formation to afford the fullerenyl monoanion **INT-1** bearing a heterocycle fused with a [5,6]-junction, and final intramolecular cyclization of the nearby anionic fullerenyl carbon with another –CH_2_Br group to provide product **2** with an unprecedented 1,2,4,17-addition pattern. Product **2** has a carbocycle fused to a [6,6]-junction and a heterocycle fixed to a [5,6]-junction at the *cis*-3′ site (Fig. 1c). Density functional theory (DFT) calculations at the B3LYP/6-31G(d) level^15^ indicate that **INT-1** is preferably formed and that the desired product **2** is most likely generated because it is more stable than other two possible isomers **2′** and **2′′** by at least 18.9 kcal mol^–1^ (Fig. 2) (see ESI† for details).

**Scheme 1:**
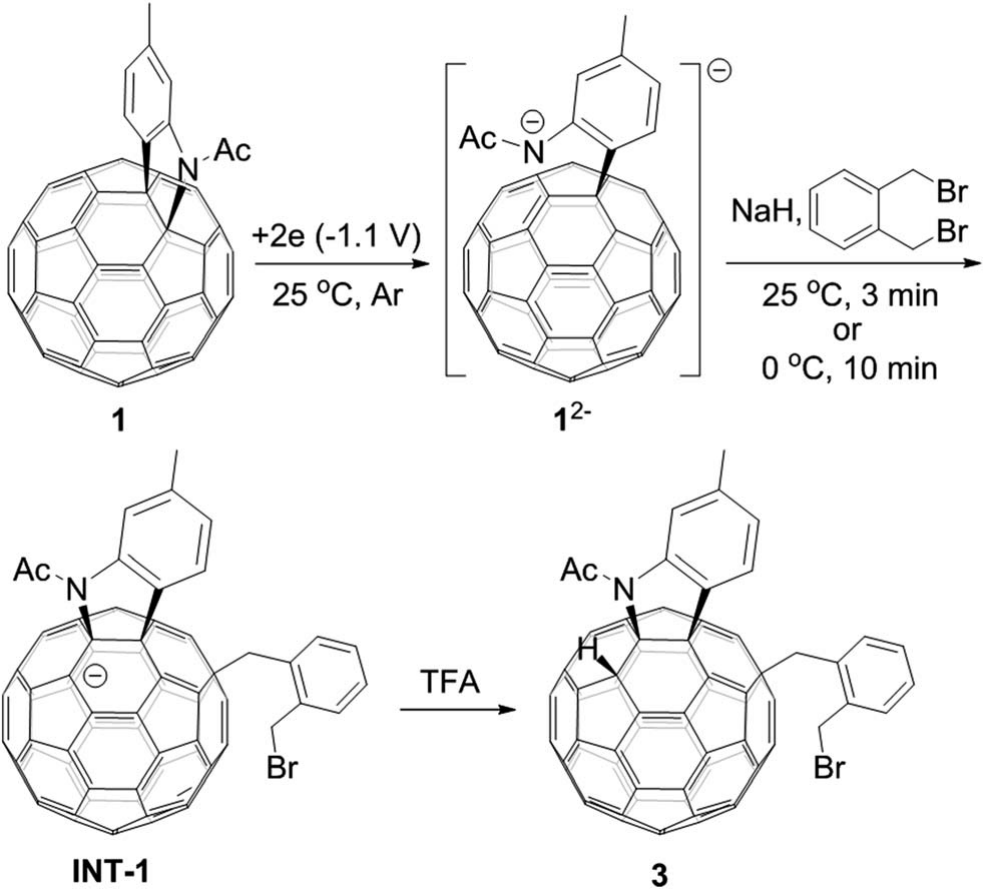
Reaction of **1^2–^** with 1,2-bis(bromomethyl)benzene at 25 °C for 3 min or 0 °C for 10 min and subsequent quenching with TFA.

[60]Fulleroindoline **1** was synthesized according to our previous procedure.^16^ To testify our assumption, we performed the reaction of **1^2–^**, which was generated from neutral **1** in *ortho*-dichlorobenzene (*o*-DCB) solution containing 0.1 M tetra-*n*-butylammonium perchlorate (TBAP) by controlled potential electrolysis (CPE) at –1.1 V,7*b* with 10 equiv. of 1,2-bis(bromomethyl)benzene and 10 equiv. of NaH solution under an argon atmosphere. NaH was added to the reaction system in order to remove the trace amount of residual water and further reduce byproduct formation.^17^ We found that when the reaction was allowed to proceed at 25 °C for 3 min and then immediately quenched with 2 equiv. of trifluoroacetic acid (TFA), 1,2,3,16-adduct **3** was obtained in 48% yield. The yield of **3** could be further increased to 58% if the reaction was conducted at 0 °C for 10 min under otherwise same conditions (Scheme 1).

**Scheme 2:**
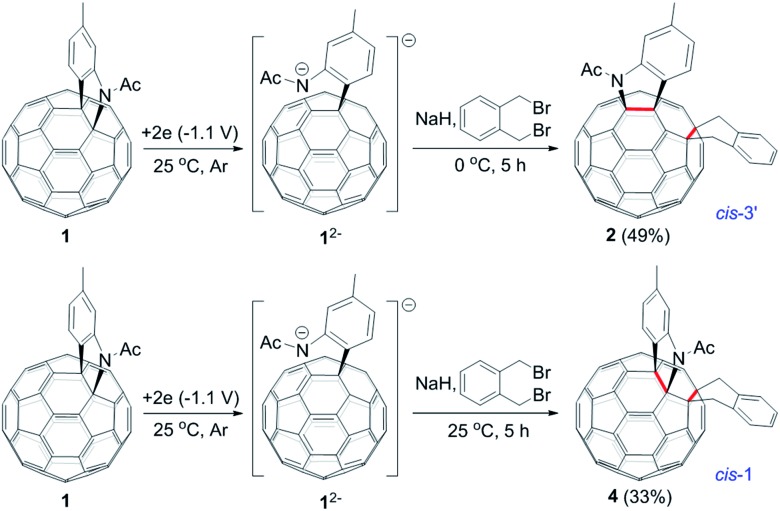
Reaction of **1^2–^** with 1,2-bis(bromomethyl)benzene at 0 °C and 25 °C to afford **2** and **4**, respectively.

The isolation of **3** indicated that the proposed intermediate **INT-1** was indeed formed. Surprisingly, it was found that further reaction at 25 °C and 0 °C for 5 h afforded totally different products, exhibiting temperature-controlled regioselectivity (Scheme 2). When the reaction was performed at 0 °C, the desired product **2** could be generated in 49% yield. Product **2** has an unprecedented 1,2,4,17-addition pattern, which can also be named as *cis*-3′ isomer and possesses a carbocycle fused to a [6,6]-junction and a heterocycle connected to a [5,6]-junction (see Fig. 1c). For a bis-cycloadduct, the two cycles are usually attached to two [6,6]-junctions of C_60_.^2^,^3^ To the best of our knowledge, there has been no precedent with the two cycles bonded to a [6,6]-junction and a [5,6]-junction, respectively, for a non-tethered bis-cycloadduct.^18^ In sharp contrast, another regioisomer **4** with the 1,2,3,4-addition pattern, also named as *cis*-1 isomer where both the carbocycle and heterocycle are fused to two neighboring [6,6]-junctions of C_60_ (see Fig. 1b), could be generated in 33% yield at 25 °C. It should be mentioned that part of **2** decomposed when it was purified on a silica gel column at 25 °C, and the isomerized product **4** could be isolated. This phenomenon was not observed when the purification process was performed at 0 °C.

Theoretical calculations showed that *cis*-3′ isomer **2** was less stable than *cis*-1 isomer **4** by 14.2 kcal mol^–1^ at the B3LYP/6-31G(d) level (see ESI† for details). Although **2** was not very stable, it remained nearly unchanged after being stirred in pure *o*-DCB at ambient temperature for 5 h. However, **2** tended to decompose to generate isomeric **4** in a low yield of 13% and some unidentified residue along with 21% of recovered **2** when stirred in *o*-DCB containing NaH and 0.1 M TBAP at room temperature for 5 h.

**Scheme 3:**
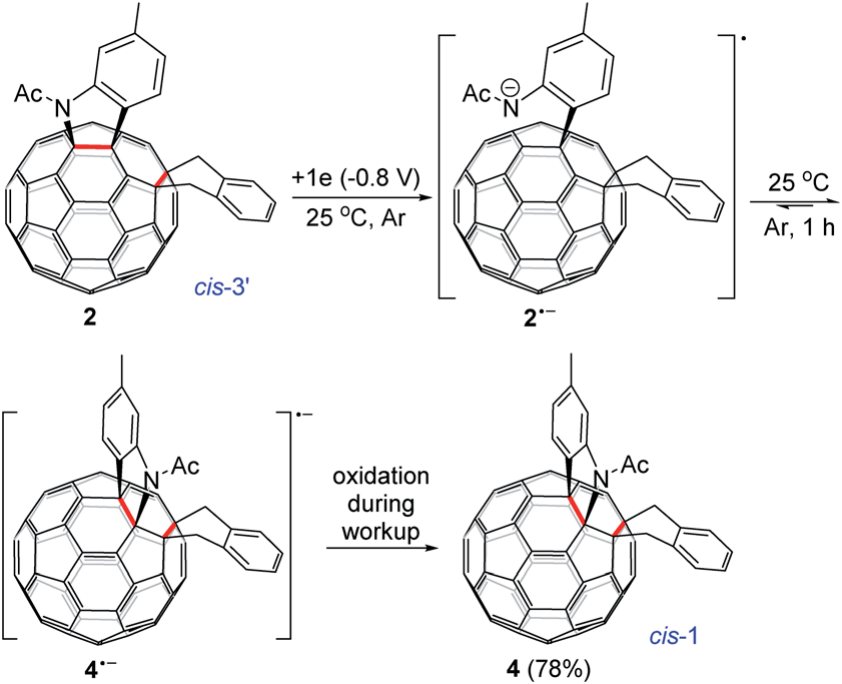
Electrochemical rearrangement of **2** to **4** at 25 °C.

Monitoring of the reaction of **1^2–^** with 1,2-bis(bromomethyl)benzene at 25 °C showed that *cis*-3′ isomer **2** was initially formed and then gradually converted to *cis*-1 isomer **4**, indicating that **2** was the precursor of **4** under our electrochemical conditions at 25 °C. Control experiments precluded the thermal rearrangement of **2** to **4** at 25 °C as the predominant process due to the low efficiency and poor yield (*vide supra*). The cyclic voltammogram (CV) of **2** exhibited irreversible first redox process of **2** (Fig. S3†) and more positively shifted than that of **1** (Table S1†).7*b* This CV result suggested that the formed **2** could be reduced *in situ* by the unreacted **1^2–^** to produce radical anions **1˙^–^** and **2˙^–^** during the reaction. On the other hand, after completion of the reaction, **2** could also be reduced to **2˙^–^** by the negatively charged system generated by CPE. Radical anion **2˙^–^** might undergo rearrangement to provide **4˙^–^**, which was subsequently oxidized to neutral **4** by an oxidizing species such as oxygen during the workup process. This assumption was supported by a control experiment, which showed that the electrochemically generated radical monoanion **2˙^–^** by CPE from **2** indeed underwent rearrangement within 1 h to afford **4** in 78% yield at 25 °C (Scheme 3).

**Scheme 4:**
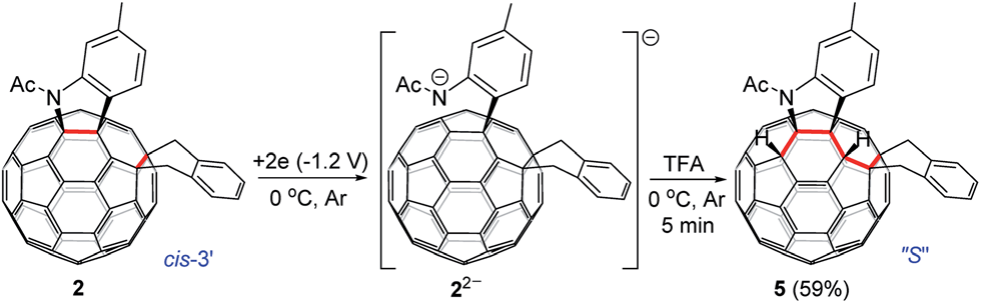
Electrochemical transformation of **2** into **5**.

As seen in the CV of **2** (Fig. S3†), the first redox process of **2** was irreversible and its second one was quasi-reversible. This result indicated that the dianionic species **2^2–^** should also have a ring-opened structure and could be employed for further functionalization. Pleasingly, it was found that the protonation of the dianionic **2^2–^** with 2 equiv. of TFA at 0 °C for 5 min afforded 1,2,3,4,9,10-adduct **5** in 59% yield (Scheme 4). The preferred formation of **5** from the protonation of **2^2–^** was also supported by theoretical calculations (see ESI† for details).

Products **2**, **3**, **4** and **5** were characterized by HRMS, ^1^H NMR, ^13^C NMR, FT-IR, UV-vis and fluorescence spectra as well as CV and DPV. Particularly, the UV-vis spectra exhibit characteristic absorption peaks and features for each type of fullerene derivatives.^2^ Products **2** and **5** are new types of fullerene derivatives with unprecedented addition patterns, thus displaying new absorption features. However, the UV-vis absorption feature of **3** was very similar to those of other 1,2,3,16-adducts,^7^ while that of **4** showed a diagnostic spike at 431 nm, pretty close to those of other 1,2,3,4-adducts.^4^ The observed similar absorption features of **3** and **4** to those reported in the literature confirmed their structural assignments. The fluorescence spectra of bis-cycloadducts **2** and **4** in chloroform solution with an excitation wavelength of 550 nm resembled each other and exhibited two peaks at ∼720 and ∼800 nm, while those of **3** and **5** showed two peaks at 711 and 790 nm for the former and 696 and 766 nm for the latter, respectively. Two similar fluorescence peaks have also been observed for bis-cycloadducts of C_60_ in the literature.^19^ The CVs and DPVs of tetra-functionalized products **2–4** in the range of 0 to –2.0 V showed predominantly four redox processes, whereas those of hexa-functionalized product **5** exhibited only three redoxes in the same range probably due to its decreased electron-accepting ability. The first and second redox waves of **4** were reversible, while the first redox of **3** was quasi-reversible. In contrast, both **2** and **5** showed an irreversible wave for the first redox process. These results hint that compounds **2**, **3** and **5** may be further functionalized by the electrochemical reduction to their monoanionic and/or dianionic stages.

**Fig. 3:**
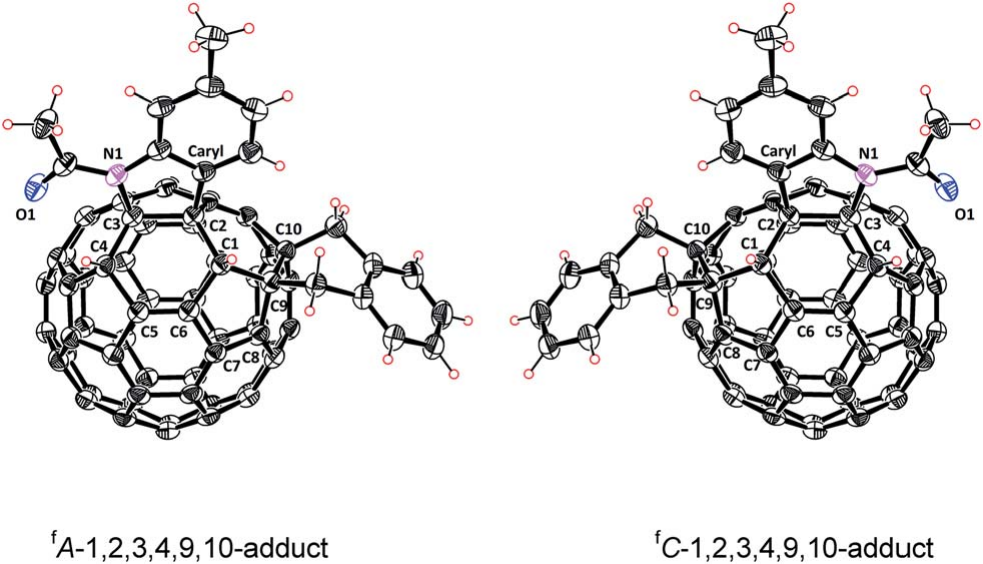
ORTEP diagram of two enantiomers of **5** with 30% thermal ellipsoids. Solvent molecules are omitted for clarity.

In addition, the assigned structure of **5** was unequivocally confirmed by its single-crystal X-ray crystallography (Fig. 3).^20^ As a consequence of the inherent chirality possessed by **5**, the crystal structure contains a pair of enantiomers, and they are in equal amounts, which is encountered in other fullerene crystals.6f,7c The structure of **5** reveals clearly that a heterocycle is bonded to C_60_ through a C_aryl_ atom and a N atom at C2 and C3 sites, respectively, and that a carbocycle is bonded to C_60_ through two benzyl groups at the C9 and C10 sites, respectively. Two hydrogen atoms are attached to C1 and C4, neighboring the heterocycle. The six-functionalized carbon atoms of the fullerene cage are uplifted from the spherical surface notably because of their sp^3^ characters. The bond lengths for C1–C2, C2–C3, C3–C4, C1–C9, C9–C10 and C10–C11 are 1.580(2) Å, 1.642(2) Å, 1.557(2) Å, 1.622(3) Å, 1.596(2) Å, and 1.522(2) Å, respectively, and are within the range of typical C–C single bond lengths. In comparison, C5–C6 has a bond length of 1.351(2) Å, thus possessing double bond character.7*b* The single-crystal structure of **5** clearly demonstrates that it has a unique “*S*”-shaped configuration.

## Conclusions

In summary, we have achieved the efficient and regioselective synthesis of the tetra-functionalized 1,2,4,17-adduct and the hexa-functionalized 1,2,3,4,9,10-adduct of C_60_. The 1,2,4,17-adduct has an unprecedented *cis*-3′ addition pattern, meanwhile the 1,2,3,4,9,10-adduct exhibits a unique “*S*”-shaped addition pattern. Both of them bear a carbocycle fused to a [6,6]-junction and a heterocycle fixed to a [5,6]-junction. The reaction of the electrochemically generated dianonic [60]fulleroindoline with 1,2-bis(bromomethyl)benzene for a short time and subsequent acid quenching afford the expected 1,2,3,16-adduct, proving our assumed addition site in the first step. Further reaction without acid quenching provides products with different addition patterns depending critically on the reaction temperature. The product obtained at 0 °C for 5 h is the desired unprecedented *cis*-3′ adduct. In contrast, the same reaction at 25 °C for 5 h selectively affords the more stable *cis*-1 isomer, which turns out to be generated by the rearrangement of the *cis*-3′ isomer induced by the negatively charged system. Intriguingly, the obtained *cis*-3′ adduct can be further regioselectively transformed into the “*S*”-shaped hexa-functionalized product. The observed high regioselectivities are controlled by charge distribution, steric effect and reaction temperature. It is anticipated that further chemical or electrochemical manipulations of the tetra- and hexa-functionalized [60]fullerene derivatives would provide new fullerene derivatives with novel addition patterns and physical properties.

## Conflicts of interest

The authors declare no conflict of interest.

## Supplementary Material

Supplementary information

Crystal structure data
