# Supplementary material for: Regioselective electrosynthesis of tetra- and hexa-functionalized [60]fullerene derivatives with unprecedented addition patterns
Source: Chem Sci. 2019 Nov 20;11(2):384–8. doi: 10.1039/c9sc02131k (PMC7021186; doi:10.1039/c9sc02131k)
Supplement: Supplementary file 1 [file SC-011-C9SC02131K-s001.pdf]

Electronic Supplementary Material (ESI) for Chemical Science.
This journal is © The Royal Society of Chemistry 2019

Electronic Supplementary Information

Regioselective Electrosynthesis of Tetra- and Hexa-functionalized [60]Fullerene Derivatives with Unprecedented Addition Patterns

Kai-Qing Liu,[‡,a] Jun-Jie Wang,[‡,a] Xing-Xing Yan,[a] Chuang Niu[a] and Guan-Wu Wang*[,a,b]

[a] *Hefei National Laboratory for Physical Sciences at Microscale, CAS Key Laboratory of Soft Matter Chemistry, iChEM (Collaborative Innovation Center of Chemistry for Energy Materials), Center for Excellence in Molecular Synthesis of CAS, and Department of Chemistry, University of Science and Technology of China, Hefei, Anhui 230026, P. R. China E-mail: gwang@ustc.edu.cn; Fax: +86 551 3607864; Tel: +86 551 3607864*

[b] *State Key Laboratory of Applied Organic Chemistry, Lanzhou University, Lanzhou, Gansu 730000, P. R. China*

[‡] *These authors contributed equally to this work.*

Table contents

Potential Energy Surface Study on the Ring-Closure Process for **INT-1**	S2
Experimental Procedures and Characterization Data of **2**–**5**	S3-S5
HPLC Analysis of the Crude Products Obtained from the Reaction of $\mathbf{1}^{2-}$ with 1,2-Bis(bromomethyl)benzene	S6
Cyclic Voltammograms and Differential Pulse Voltammograms of Compounds **1**–**5**	S7-S8
Optimized Structures and Relative Calculated Energies of **2** and **4**	S8
Theoretical Study on the Formation of **5** from **2** *via* Electrochemical Protonation	S9-S11
Single-Crystal X-Ray Crystallography of Compound **5**	S12-S13
UV–Vis Spectra of Compounds **2**–**5**	S14
Fluorescence Spectra of Compounds **2**–**5**	S15
^{1}H NMR Spectrum of Compound **2**	S16
^{1}H NMR Spectrum of Compound **3**	S17
^{13}C NMR Spectrum of Compound **3**	S18
^{1}H NMR Spectrum of Compound **4**	S19
^{13}C NMR Spectrum of Compound **4**	S20
^{1}H NMR Spectrum of Compound **5**	S21
^{13}C NMR Spectrum of Compound **5**	S22
The xyz Coordinates and Energies for the Lowest Energy Structures of **I**–**VII**, **2**, **2'**, **2''**, and **4**	S23-S48
References	S48

Potential Energy Surface Study on the Ring-Closure Process for INT-1

Potential energy surface (PES) scan gives a reasonable prediction of the ring-closure position.[1] To gain insight into the reaction pathway, we performed the relaxed PES scans about the dihedral angel of C1−C2−C3−C4 (χ) (Figure S1) at the B3LYP/6-31G(d) level, which predicted the preferred ring-closure process for the formation of **INT-1**. According to our previous work,[2] the first benzyl group of 1,2-bis(bromomethyl)benzene would attack at the *para*-position of the aryl addend to generate structure **A** with a χ of −11.1°. When the scan was clockwise, an energy barrier of 2.7 kcal/mol was required for the rotation from structure **A** to structure **B** with a χ of −68.1°. The potential energy would decrease until the dihedral angle χ reached to −117.1° (structure **C**), where the nitrogen atom was located above the [5,6]-junction and would not automatically lead to the C−N bond formation. Then, an energy barrier of 12.3 kcal/mol was required when the attached aryl group continued to rotate clockwisely to structure **D** with a χ of −203.8°. Subsequently, when the aryl group was rotated until above the [6,6]-junction, resulting in the cyclization automatically to form structure **E** with a χ of −218.7° and a relatively low energy of −12.1 kcal/mol. Finally, structure **E** further rotated to the slightly more stable structure **H** with a χ of −236.7° and a relative energy of −12.6 kcal/mol. On the other hand, when the scan from structure **A** was performed anticlockwisely until above the [5,6]-junction, cyclization would occur simultaneously via the C_{60}−N bond formation to provide structure **F** (also **INT-1**) with a χ of 0.0°, which had the lowest energy of −22.7 kcal/mol in the overall energy profile. A high energy barrier of 27.9 kcal/mol was demanded for the C_{60}−N bond rupture and subsequent anticlockwise rotation of the aryl group towards structure **G**. Further anticlockwise rotation would eventually provide structure **H**. As seen from the overall energy profile, **INT-1** (also **F**) would be preferably formed. Therefore, the intramolecular cyclization of **INT-1** with the release of Br^- would afford the observed kinetic product **2**, fully agreeing with the experimental result.

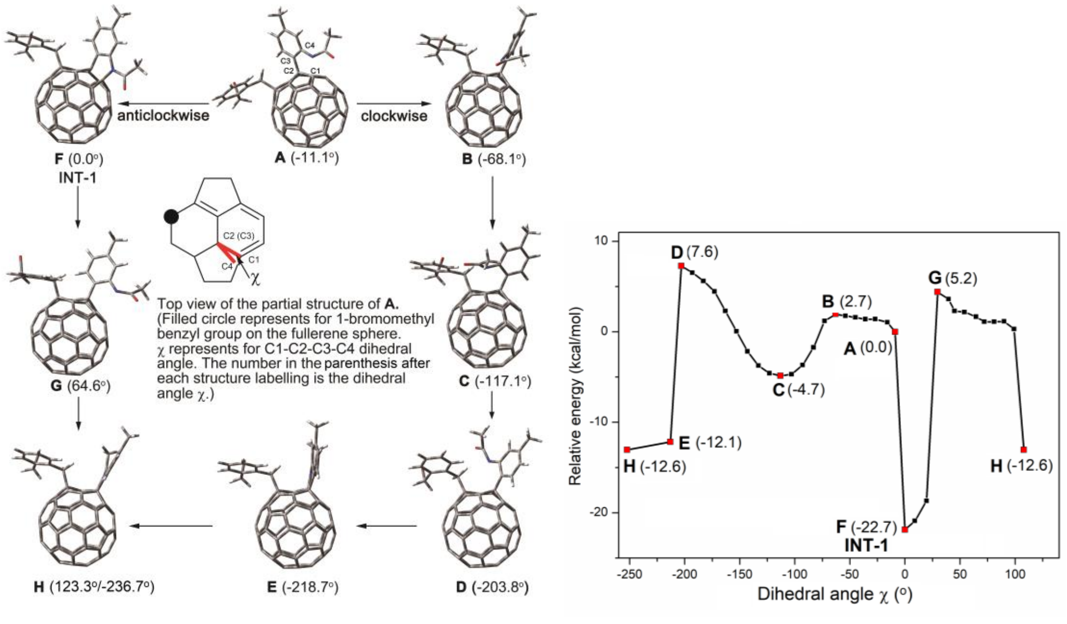

Figure S1. Potential energy surface scans and energy profile about dihedral angel χ.

Experimental Procedures and Characterization Data of 2–5

General Procedures. All electrochemical measurements and reactions were performed under an argon atmosphere using a SHANGHAI CHENHUA CHI630D workstation. Tetra-*n*-butylammonium perchlorate (TBAP) was recrystallized from absolute ethanol and dried in a vacuum at 313 K prior to use. Other chemicals were obtained commercially and used without further purification. Controlled potential electrolysis (CPE) was carried out on a potentiostat/galvanostat using an "H"-type cell which consisted of two platinum gauze electrodes (serving as working and counter electrodes, respectively) separated by a sintered glass frit. A conventional three-electrode cell was used for cyclic voltammogram (CV) and differential pulse voltammogram (DPV) measurements and consisted of a 2-mm diameter platinum disc working electrode, a platinum counter electrode, and a saturated calomel reference electrode (SCE). The SCE was separated from the bulk of the solution by a fritted-glass bridge of low porosity which contained the solvent/supporting electrolyte mixture.

Cyclic Voltammograms and Differential Pulse Voltammograms. Compounds **2**–**5** dissolved in anhydrous *ortho*-dichlorobenzene (*o*-DCB) (c = 1.0×10^{-3} M) containing 0.1 M TBAP were added into an electrochemical cell under an argon atmosphere at room temperature. The CV measurements were then undertaken at a scan rate of 20 mV/s.

Synthesis of Compound 3. 17.1 mg (0.020 mmol) of **1** was electroreduced by CPE at –1.10 V vs SCE in 15.8 mL of *o*-DCB solution containing 0.1 M TBAP under an argon atmosphere at room temperature. The potentiostat was turned off when the theoretical number of coulombs required for full conversion of **1** into $\mathbf{1}^{2-}$ was reached. Then, 8.0 mg of NaH (57–63% oil dispersion, 0.200 mmol) and 53.3 mg of 1,2-bis(bromomethyl)benzene (0.200 mmol) were sequentially added to the solution of $\mathbf{1}^{2-}$ at 0 °C, and the reaction mixture was stirred for 10 min and then quenched by TFA (3.0 μL, 0.040 mmol). The reaction mixture was then filtered through a silica gel plug to remove TBAP. After evaporation in vacuo, the residue was separated on a silica gel column with CS_2/CH_2Cl_2 (6:1) as the eluent to afford **3** (12.0 mg, 58%) as an amorphous brown solid and recovered **1** (2.6 mg, 15%). ^{1}H NMR (400 MHz, $CDCl_3$) δ 8.22 (d, J = 7.8 Hz, 1H), 7.43–7.32 (m, 4H), 7.27–7.22 (m, 2H), 6.00 (s, 1H), 4.65 (d, J = 10.3 Hz, 1H), 4.60 (d, J = 10.3 Hz, 1H), 4.56 (d, J = 13.7 Hz, 1H), 4.45 (d, J = 13.7 Hz, 1H), 2.84 (s, 3H), 2.62 (s, 3H); ^{13}C NMR (101 MHz, $CDCl_3$) δ 168.64 (*C*=O), 156.65, 153.91, 151.77, 149.83, 149.65, 149.23, 148.92, 148.44, 148.38, 148.08, 147.98, 147.71, 147.67, 147.30, 147.17, 147.01, 146.88, 146.52, 146.49, 146.36, 146.20, 146.07, 145.97, 145.93, 145.44, 145.36, 145.20, 144.83, 144.77, 144.75, 144.68, 144.45, 144.39, 144.29, 143.99, 143.60, 143.49, 143.23, 143.18, 142.74, 142.47, 142.24, 142.03, 141.99, 141.72, 141.63, 141.48, 141.29, 141.23, 140.64, 140.39, 140.31, 140.09, 138.56, 137.32, 136.84, 136.42, 134.43, 134.40, 133.17, 131.15, 129.05, 128.91, 128.70, 128.25, 126.09, 125.91, 115.67,

83.89 (sp^3-*C* of C_{60}), 63.00 (sp^3-*C* of C_{60}), 58.46 (sp^3-*C* of C_{60}), 56.78 (sp^3-*C* of C_{60}), 45.03, 32.81, 27.24, 22.28; UV–vis ($CHCl_3$) λ_{max}/nm (log ε): 251 (4.89), 286.5 (4.62), 330 (4.36), 447 (3.54), 524 (3.11), 5.93 (2.85), 690 (2.48); FT-IR ν/cm^{-1} (KBr): 2917, 2854, 1668, 1499, 1431, 1367, 1340, 1299, 1245, 1192, 1085, 1034, 956, 857, 810, 795, 761, 617, 603, 566, 529; MALDI-TOF MS m/z calcd for $C_{77}H_{18}ONBr$ $[M]^+$ 1051.0566, found 1051.0563.

Synthesis of Compound 2. 17.2 mg (0.020 mmol) of **1** was electroreduced by CPE at –1.10 V vs SCE in 15.8 mL of *o*-DCB solution containing 0.1 M TBAP under an argon atmosphere at room temperature. The potentiostat was turned off when the theoretical number of coulombs required for full conversion of **1** into **1**$^{2-}$ was reached. Then, 8.0 mg of NaH (57–63% oil dispersion, 0.200 mmol) and 54.4 mg of 1,2-bis(bromomethyl)benzene (0.200 mmol) were sequentially added to the solution of **1**$^{2-}$ at 0 °C, and the reaction mixture was stirred for 5 h. The reaction mixture was then filtered through a silica gel plug to remove TBAP. After evaporation in vacuo, the residue was separated on a silica gel column with CS_2/CH_2Cl_2 (6:1) as the eluent at 0 °C to afford **2** (9.4 mg, 49%) as an amorphous brown solid and recovered **1** (2.2 mg, 13%). ^{1}H NMR (400 MHz, CS_2/DMSO-d_6) δ 8.35 (d, *J* = 7.9 Hz, 1H), 7.42–7.31 (m, 5H), 7.13 (d, *J* = 7.9 Hz, 1H), 4.77 (d, *J* =14.2 Hz, 1H), 4.67 (d, *J* = 14.1 Hz, 1H), 4.09 (d, *J* =14.1 Hz, 1H), 3.46 (d, *J* =14.2 Hz, 1H), 2.60 (s, 3H), 2.50 (s, 3H); ^{13}C NMR spectrum with good signal-to-noise ratio could not be obtained due to the instability and low solubilty of **2**. UV–vis ($CHCl_3$) λ_{max} nm (log ε) 248 (5.09), 304.5 (4.73), 352 (4.45), 416 (3.97), 493 (3.69), 581 (3.19), 652 (2.98), 762 (2.70); FT-IR ν/cm^{-1} (KBr) 2920, 2851, 1667, 1499, 1449, 1429 1366, 1337, 1299, 1246, 1031, 748, 685, 602, 524; ESI MS *m/z* calcd for $C_{77}H_{18}ON$ $[M+H]^+$ 972.1383, found 972.1376.

Synthesis of Compound 4. 17.1 mg (0.020 mmol) of **1** was electroreduced by CPE at –1.10 V vs SCE in 15.8 mL of *o*-DCB solution containing 0.1 M TBAP under an argon atmosphere at room temperature. The potentiostat was turned off when the theoretical number of coulombs required for full conversion of **1** into **1**$^{2-}$ was reached. Then, 8.0 mg of NaH (57–63% oil dispersion, 0.200 mmol) and 53.3 mg of 1,2-bis(bromomethyl)benzene (0.200 mmol) were sequentially added to the solution of **1**$^{2-}$ at 25 °C, and the reaction mixture was stirred for 5 h. The reaction mixture was then filtered through a silica gel plug to remove TBAP. After evaporation in vacuo, the residue was separated on a silica gel column with CS_2/CH_2Cl_2 (6:1) as the eluent to afford **4** (6.3 mg, 33%) as an amorphous brown solid and recovered **1** (2.1 mg, 12%). ^{1}H NMR (400 MHz, $CDCl_3$) δ 8.07 (d, *J* = 7.7 Hz, 1H), 7.53 (d, *J* = 7.1 Hz, 1H), 7.43 (s, 1H), 7.41–7.26 (m, 4H), 4.31 (d, *J* = 13.8 Hz, 1H), 4.13 (d, *J* = 13.8 Hz, 1H), 4.03 (s, 2H), 2.76 (s, 3H), 2.60 (s, 3H); ^{13}C NMR (101 MHz, $CDCl_3$, with $Cr(acac)_3$ as relaxation reagent) δ 170.06 (*C*=O), 153.32, 150.24, 149.58, 149.28, 149.17, 148.60, 148.42, 147.88, 147.45, 147.39, 147.28, 147.01, 146.99, 146.90, 146.69, 146.53, 146.43, 146.13, 145.85, 145.64, 145.43, 145.21, 145.16, 145.07, 144.87, 144.82, 144.78, 144.76, 144.66, 144.53, 144.44, 144.38, 144.23, 144.02, 143.82, 143.46, 143.36, 143.12, 142.98, 142.88, 142.68, 142.44, 142.41, 142.29,

141.90, 141.52, 141.47, 141.02, 140.96, 140.88, 139.41, 139.05, 136.45, 136.21, 135.91, 135.05, 133.53, 131.92, 129.03, 127.63, 127.38, 126.67, 126.55, 125.80, 118.26, 88.02 (sp^3-*C* of C_{60}), 68.81 (sp^3-*C* of C_{60}), 64.72 (sp^3-*C* of C_{60}), 64.31 (sp^3-*C* of C_{60}), 46.05, 40.00, 25.70, 22.05; UV–vis ($CHCl_3$) λ_{max}/nm (log ε) 255 (4.97), 297 (4.60), 335 (4.39), 431 (3.65); FT-IR ν/cm^{-1} (KBr) 2919, 2850, 1665, 1596, 1498, 1433, 1363, 1331, 1294, 1248, 1053, 874, 810, 750, 567, 527; MALDI-TOF MS *m/z* calcd for $C_{77}H_{17}ON$ $[M]^+$ 971.1305, found 971.1318.

Electrochemical Conversion of 2 to 4. 6.5 mg (0.007 mmol) of **2** was electroreduced by CPE at –0.8 V vs SCE in 15.8 mL of *o*-DCB solution containing 0.1 M TBAP under an argon atmosphere at 25 ^{o}C. The potentiostat was turned off when the theoretical number of coulombs required for full conversion of **2** into $\mathbf{2}^{\bullet-}$ was reached. The reaction mixture was stirred for 1 h and subsequently oxidized by exposure to air for 0.5 h. The reaction mixture was then filtered through a silica gel plug to remove TBAP. After evaporation in vacuo, the residue was separated on a silica gel column with CS_2/CH_2Cl_2 (6:1) as the eluent to afford **4** (5.1 mg, 78%) as an amorphous brown solid.

Synthesis of Compound 5. 1.9 mg (0.002 mmol) of **2** was electroreduced by CPE at –1.20 V vs SCE in 15.8 mL of *o*-DCB solution containing 0.1 M TBAP under an argon atmosphere at 0 ^{o}C. The potentiostat was turned off when the theoretical number of coulombs required for full conversion of **2** into $\mathbf{2}^{2-}$ was reached. Then TFA (0.3 μL, 0.004 mmol) was added and the reaction mixture was stirred for 5 min. The reaction mixture was then filtered through a silica gel plug to remove TBAP. The same procedure was repeated three times. After evaporation in vacuo, the residue combined from the four runs was separated on a silica gel column with CS_2/CH_2Cl_2 (6:1) as the eluent to afford **5** (4.6 mg, 59%) as an amorphous brown solid and byproduct **4** (2.1 mg, 27%). ^{1}H NMR(400 MHz, CS_2/DMSO-d_6) δ 8.18 (brs, 1H), 7.35–7.26 (m, 5H), 7.17 (d, *J* = 8.0 Hz, 1H), 5.93 (d, *J* = 2.3 Hz, 1H), 4.89 (d, *J* = 2.3 Hz, 1H), 4.61 (d, *J* = 13.9 Hz, 1H), 4.39 (d, *J* = 13.9 Hz, 1H), 4.10 (d, *J* = 13.9 Hz, 1H), 3.67 (d, *J* = 13.9 Hz, 1H), 2.71 (s, 3H), 2.51 (s, 3H); ^{13}C NMR (101 MHz, $C_2D_2Cl_4$) δ 167.93 (*C*=O), 152.29, 151.76, 150.84, 148.17, 147.83, 147.71, 147.50, 147.40, 147.33, 147.15, 147.04, 147.01, 147.00, 146.78, 146.68, 146.61, 146.59, 146.51, 146.08, 146.04, 145.63, 145.53, 145.03, 145.01, 144.75, 144.71, 144.24, 144.13, 143.87, 143.72, 143.69, 143.59, 143.49, 143.15, 142.93, 142.58, 142.42, 141.95, 141.82, 141.78, 140.91, 139.90, 139.73, 139.60, 139.56, 139.46, 139.42, 138.95, 138.74, 137.72, 137.41, 136.08, 134.60, 134.51, 133.89, 128.09, 127.38, 127.26, 126.76, 126.47, 124.66, 124.64, 124.36, 79.43 (sp^3-*C* of C_{60}), 61.30 (sp^3-*C* of C_{60}), 60.44 (sp^3-*C* of C_{60}), 59.81 (sp^3-*C* of C_{60}), 57.28 (sp^3-*C* of C_{60}), 54.86 (sp^3-*C* of C_{60}), 45.76, 43.38, 27.00, 21.19; UV–vis ($CHCl_3$) λ_{max}/nm (log ε) 251 (5.02), 316 (4.59), 334 (4.50), 380 (4.13), 429 (3.69), 527 (3.20), 621 (2.73), 676 (2.34); FT-IR ν/cm^{-1} (KBr) 2921, 2851, 1670, 1592, 1505, 1430, 1364, 1337, 1298, 1246, 1182, 1032, 851, 808, 787, 622, 599, 526; ESI MS *m/z* calcd for $C_{77}H_{20}ON$ $[M+H]^+$ 974.1539, found 974.1513.

HPLC Analysis of the Crude Products Obtained from the Reaction of $\mathbf{1}^{2-}$ with 1,2-Bis(bromomethyl)benzene at 25 °C

HPLC analysis were performed on a Cosmosil Buckyprep column (4.6 × 250 mm) with toluene as the eluent at a flow rate of 1.0 mL/min with the detector wavelength set at 326 nm.

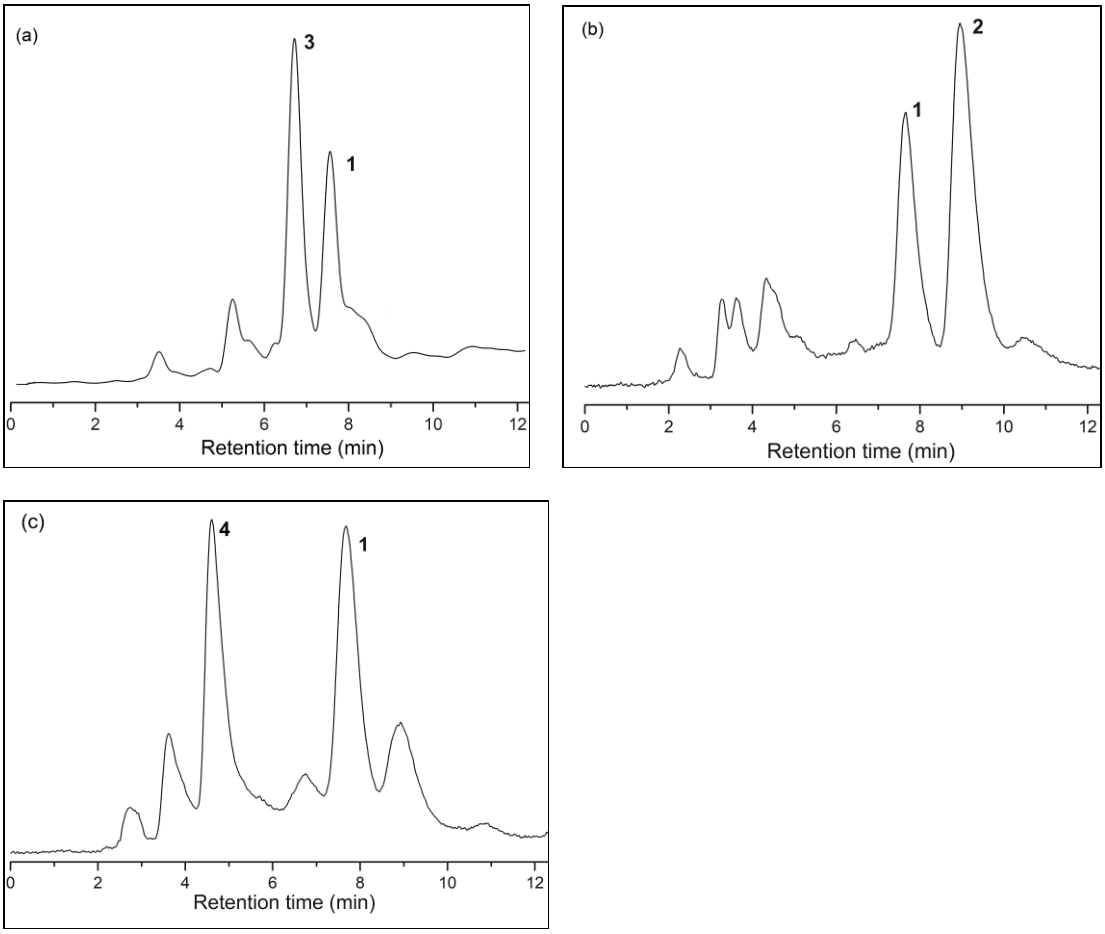

Figure S2. (a) HPLC trace of the crude products obtained from the reaction of $\mathbf{1}^{2-}$ with 1,2-bis(bromomethyl)benzene and NaH after 10 min; (b) HPLC trace of the crude products obtained from the reaction of $\mathbf{1}^{2-}$ with 1,2-bis(bromomethyl)benzene and NaH after 30 min; (c) HPLC trace of the crude products obtained from the reaction of $\mathbf{1}^{2-}$ with 1,2-bis(bromomethyl)benzene and NaH after 5 h.

Cyclic Voltammograms and Differential Pulse Voltammograms of Compounds 1–5

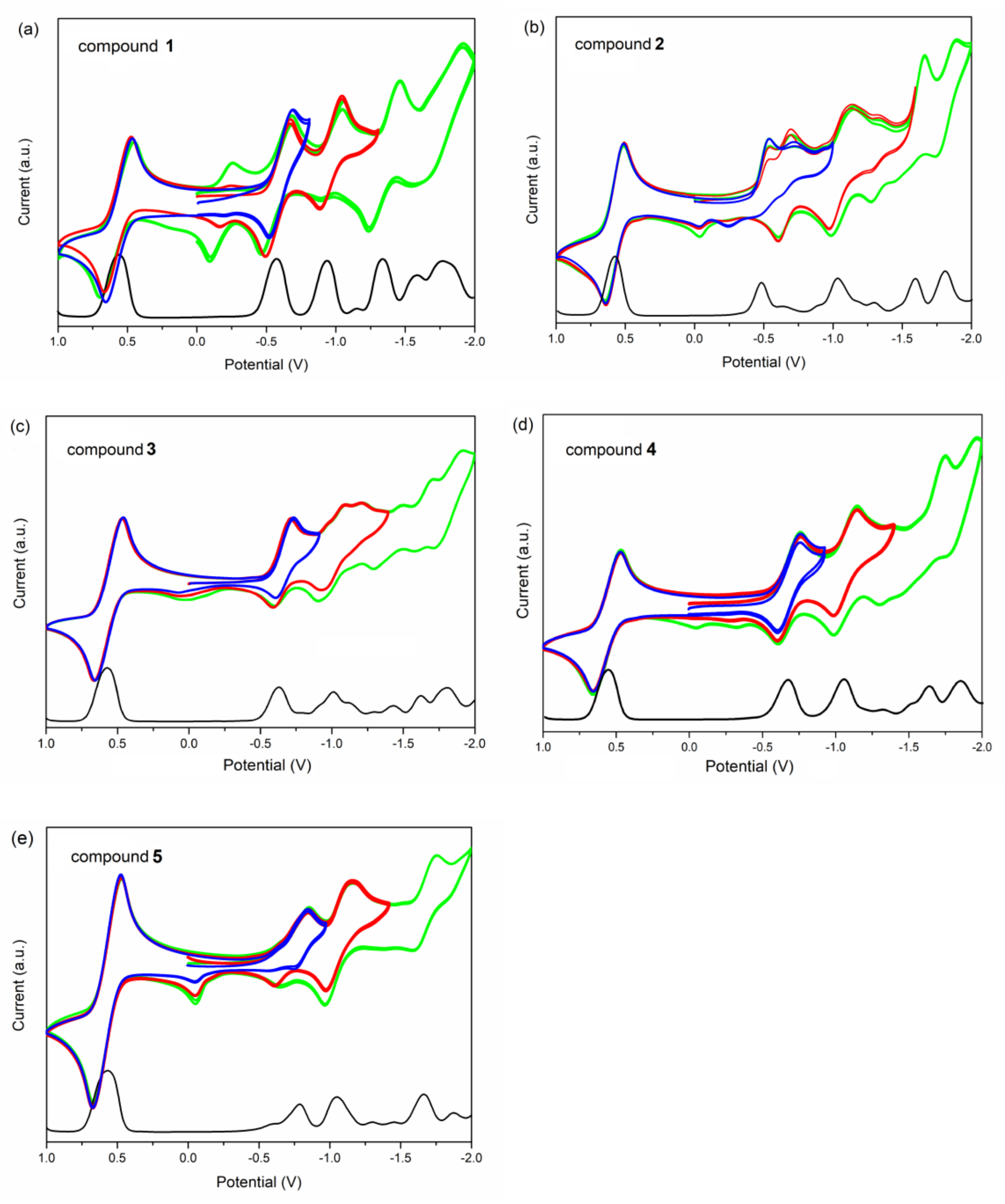

Figure S3. Cyclic voltammograms (green, red and blue line) recorded at a scan rate of 20 mV/s and differential pulse voltammograms (black line) in the potential range of 1.0 V to –2.0 V in 0.1 M solution of TBAP in *o*-DCB (ferrocene was used as an internal standard) of compounds **1–5** (1.0×10^{-3} M).

Table S1. Electrochemical Properties of Compounds 1–5

Compd	E_1/V[a]	E_2/V[a]
**1**	−0.58/−1.14	−0.94/−1.50
**2**	−0.48/−1.06	−1.03/−1.61
**3**	−0.63/−1.20	−1.01/−1.58
**4**	−0.67/−1.23	−1.05/−1.61
**5**	−0.79/−1.36	−1.05/−1.62

[a]Values were obtained from the differential pulse voltammograms versus SCE for the former and Fc/Fc^+ for the latter.

Optimized Structures and Relative Calculated Energies of 2 and 4

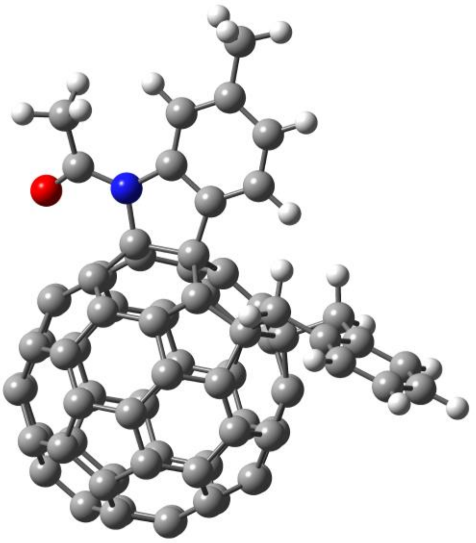

2
0.0 kcal/mol

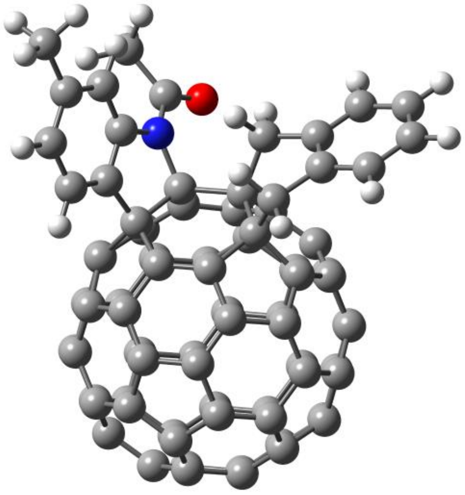

4
−14.2 kcal/mol

Figure S4. Optimized structures and relative energies (in kcal/mol) of **2** and **4** at the B3LYP/6-31G(d) level.

Theoretical Study on the Formation of 5 from 2 *via* Electrochemical Protonation

The formation of **5** from **2** by electrochemical protonation was also rationalized by theoretical calculations at the B3LYP/6-31G(d) level. The calculated natural bond orbital (NBO) charge distribution of the electrochemically generated $\mathbf{2}^{2-}$ showed that C2 (−0.078), C10 (−0.112), C29 (−0.080) and C44 (−0.092) were the four most negatively charged nonfunctionalized C_{60} carbon atoms (Figure S5). As demonstrated previously, the regioselectivity was dependent on not only the charge density distribution of the reacting species but also the stability of the formed species. In the protonation of $\mathbf{2}^{2-}$ with TFA, the most likely formed intermediates were the four anions (**I**, **II**, **III** and **IV**) generated by protonation at C2, C10, C29 and C44, respectively (Figure S6). Theoretical calculations at the B3LYP/6-31G(d) level gave a clear preference to the formation of intermediate **I** over the other three intermediates by at least 14.1 kcal/mol.

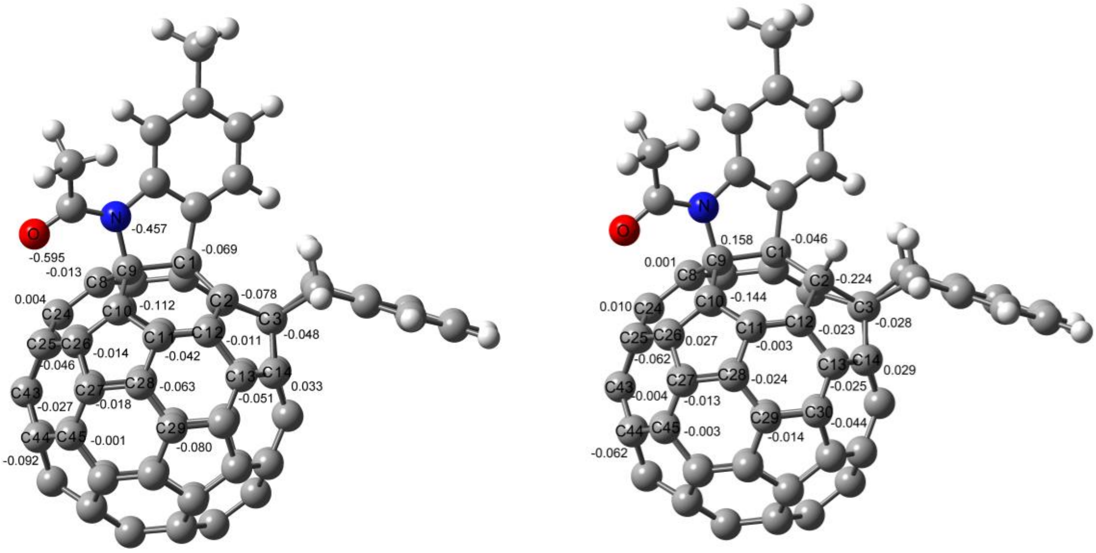

Figure S5. Partial NBO charge distribution of $\mathbf{2}^{2-}$ and **I**.

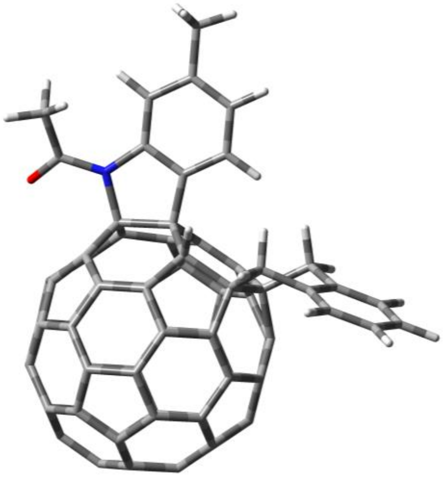

I
0 kcal/mol

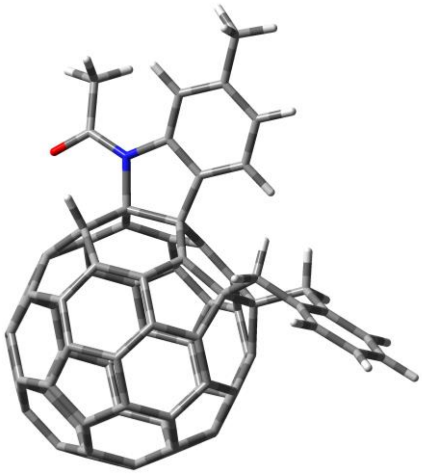

II
14.1 kcal/mol

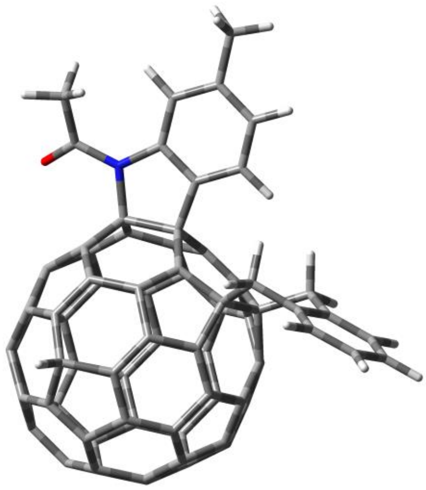

III
14.7 kcal/mol

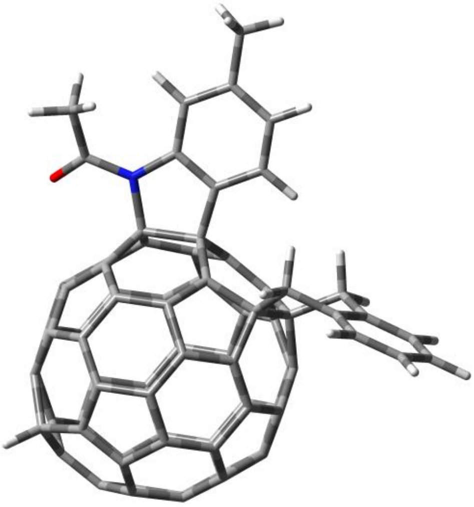

IV
18.1 kcal/mol

Figure S6. Relative energies of the optimized **I−IV** at the B3LYP/6-31G(d) level.

The calculated NBO charge distribution of intermediate **I** showed that the three most negatively charged nonfunctionalized C_{60} atoms were at C10 (−0.144), C24 (−0.062) and C42 (−0.062) (Figure S5). Similarly, the most likely sites for the second protonation would be C10, C24 and C42, affording **V**, **VI** and **VII**, respectively. Theoretical calculations at the B3LYP/6-31G(d) level demonstrated that **V** was more stable than **VI** and **VII** by at least 6.3 kcal/mol (Figure S7).

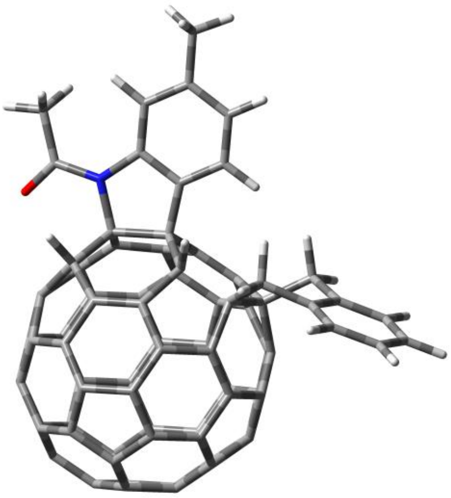

V
0.0 kcal/mol

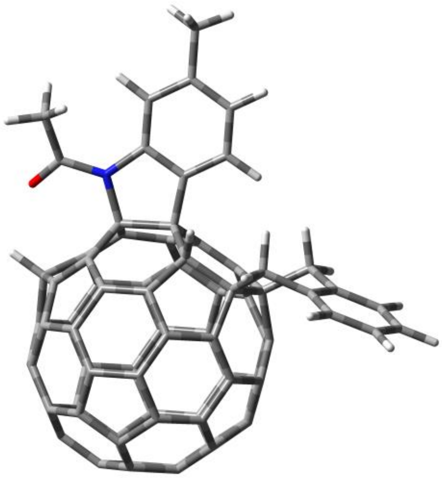

VI
6.3 kcal/mol

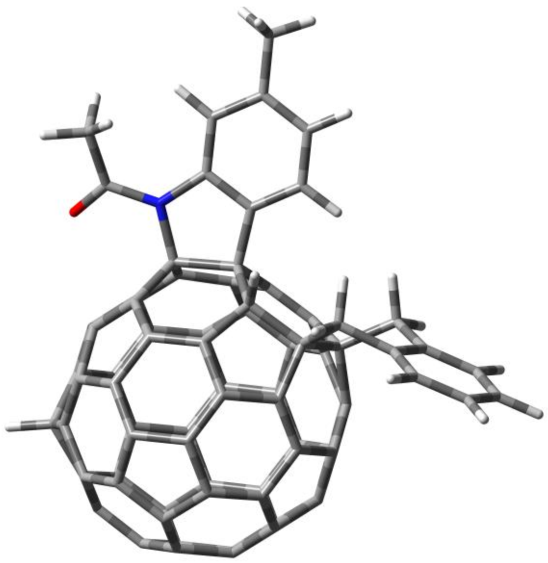

VII
18.7 kcal/mol

Figure S7. Relative energies of the optimized **V−VII** at the B3LYP/6-31G(d) level.

The above theoretical calculations indicated that **V** (also **5**) should be preferably formed from the protonation of the electrochemically generated **2**$^{2-}$ by TFA, fully consistent with the experimental result.

Single-Crystal X-Ray Crystallography of Compound 5

Black block crystals of **5** were obtained by slowly diffusing methanol into the CS_2 solution at 4 ^oC. A suitable crystal was selected and tested on a diffractometer. The crystal was kept at 291(2) K during data collection. Single-crystal X-ray diffraction data were collected on a diffractometer (Gemini S Ultra, Agilent Technologies) equipped with a CCD area detector using graphite-monochromated Cu Kα radiation (λ = 1.54184 Å) in the scan range 6.944° < 2*θ* < 140.122°. Using Olex2, the structure was solved with the ShelXS structure solution program using Direct Methods and refined with the ShelXL refinement package using Least Squares minimization. The structure was solved with direct methods using SHELXS-97 and refined with full-matrix least-squares refinement using the SHELXL-97 program within OLEX2.

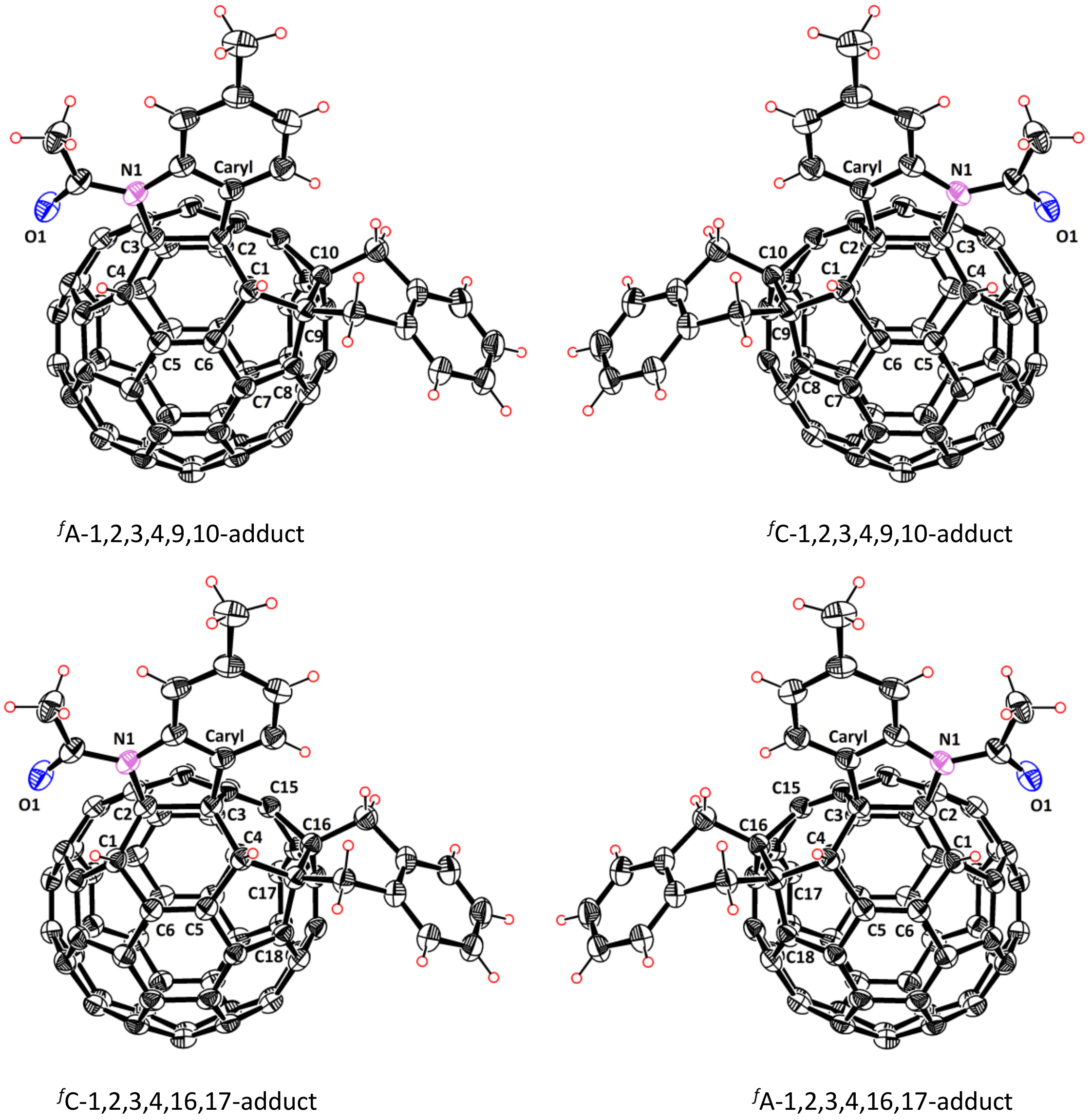

Figure S8. ORTEP diagram of the enantiomers of **5** (1:1) with 30% thermal ellipsoids. Solvent molecules are omitted for clarity.

Table S2. Crystal data and structure refinement for **5**.

Identification code	1900924
Empirical formula	$C_{77}H_{19}NO{\bullet}CS_2$
Formula weight	1050.06
Temperature/K	291(2)
Crystal system	monoclinic
Space group	$P2_1/c$
a/Å	14.2115(5)
b/Å	13.2334(4)
c/Å	26.4184(7)
α/°	90
β/°	116.384(3)
γ/°	90
Volume/Å^3	4450.9(3)
Z	4
ρ_{calc}g/cm^3	1.567
μ/mm^{-1}	1.567
F(000)	2136.0
Crystal size/mm^3	0.32 × 0.30 × 0.26
Radiation	CuKα (λ = 1.54184)
2Θ range for data collection/°	6.944 to 140.122
Index ranges	$-17 \le h \le 17$, $-10 \le k \le 15$, $-31 \le l \le 31$
Reflections collected	25299
Independent reflections	8247 [R_{int} = 0.0399, R_{sigma} = 0.0448]
Data/restraints/parameters	8247/27/751
Goodness-of-fit on F^2	1.071
Final R indexes [I >= 2σ (I)]	R_1 = 0.0789, wR_2 = 0.1994
Final R indexes [all data]	R_1 = 0.1222, wR_2 = 0.2308
Largest diff. peak/hole/e Å^{-3}	0.73/-0.78

UV–Vis Spectra of Compounds 2–5

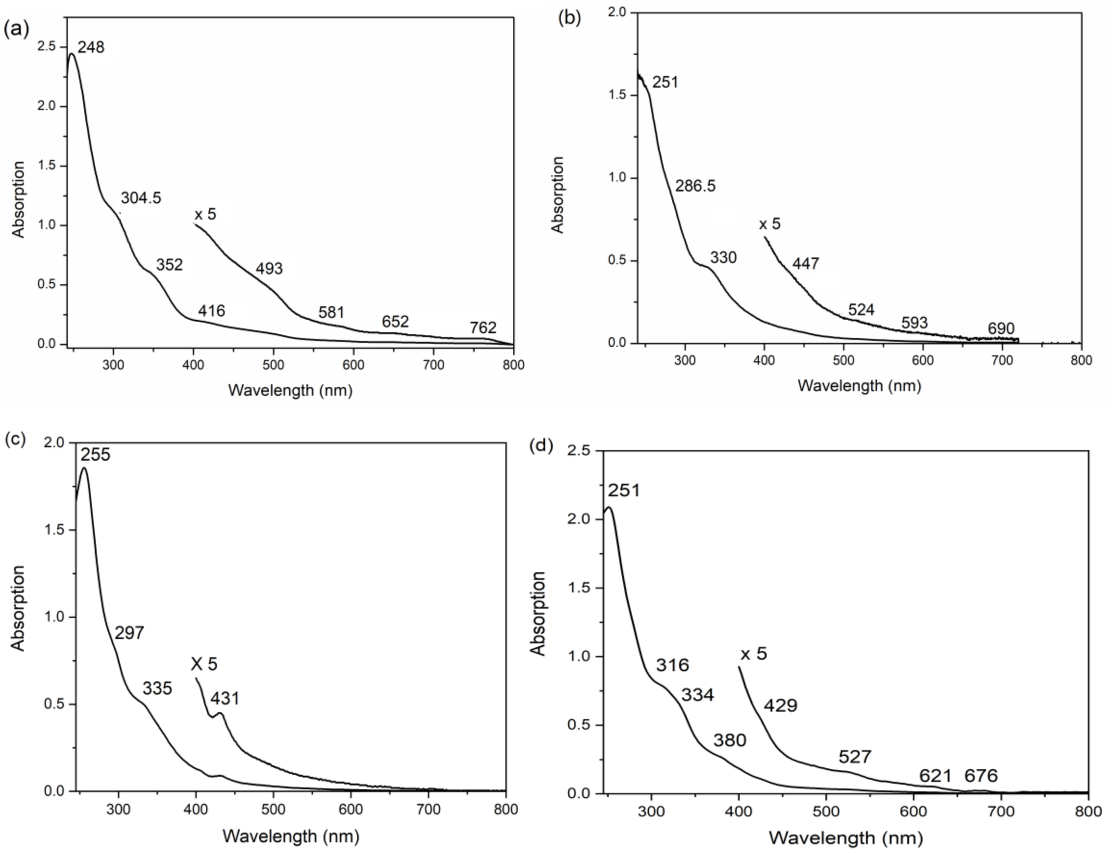

Figure S9. (a) UV–vis spectrum of compound **2** in $CHCl_3$ (2×10^{-5} M); (b) UV–vis spectrum of compound **3** in $CHCl_3$ (2×10^{-5} M); (c) UV–vis spectrum of compound **4** in $CHCl_3$ (2×10^{-5} M); (d) UV–vis spectrum of compound **5** in $CHCl_3$ (2×10^{-5} M).

Fluorescence Spectra of Compounds 2–5

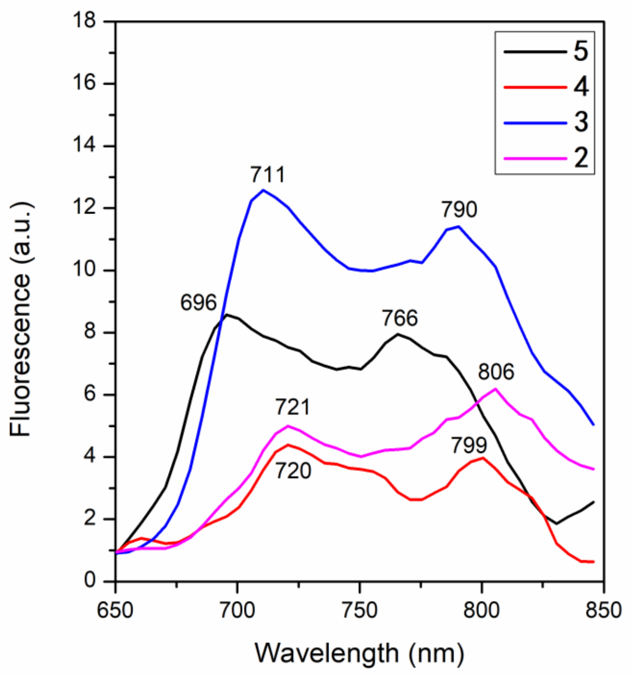

Figure S10. Fluorescence spectra of compounds **2–5** in $CHCl_3$ (4×10^{-4} M) collected from 650 to 850 nm with the excitation wavelength at 550 nm.

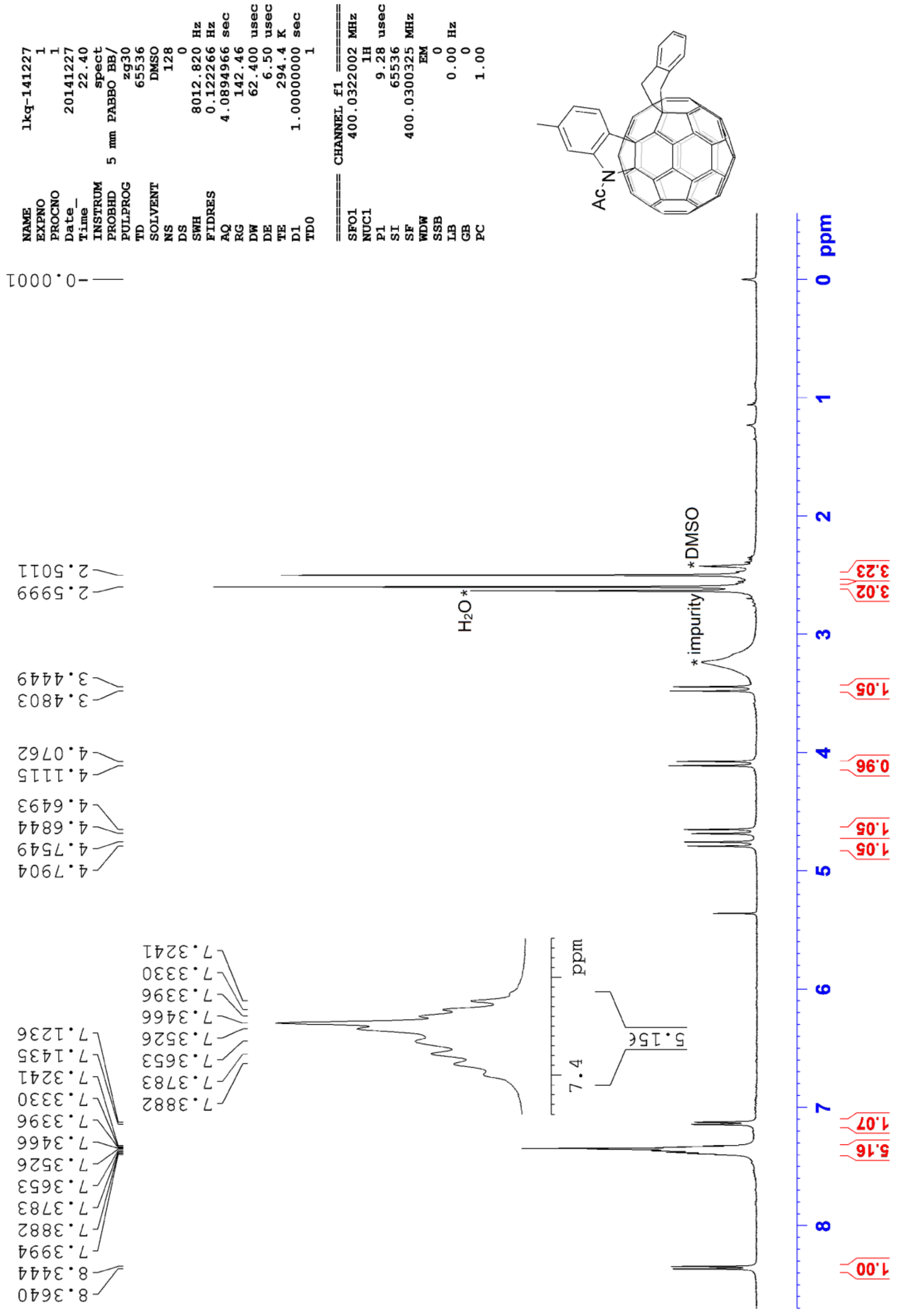

Figure S11. ^{1}H NMR (400 MHz, CS_2/DMSO-d_6) spectrum of compound **2**.

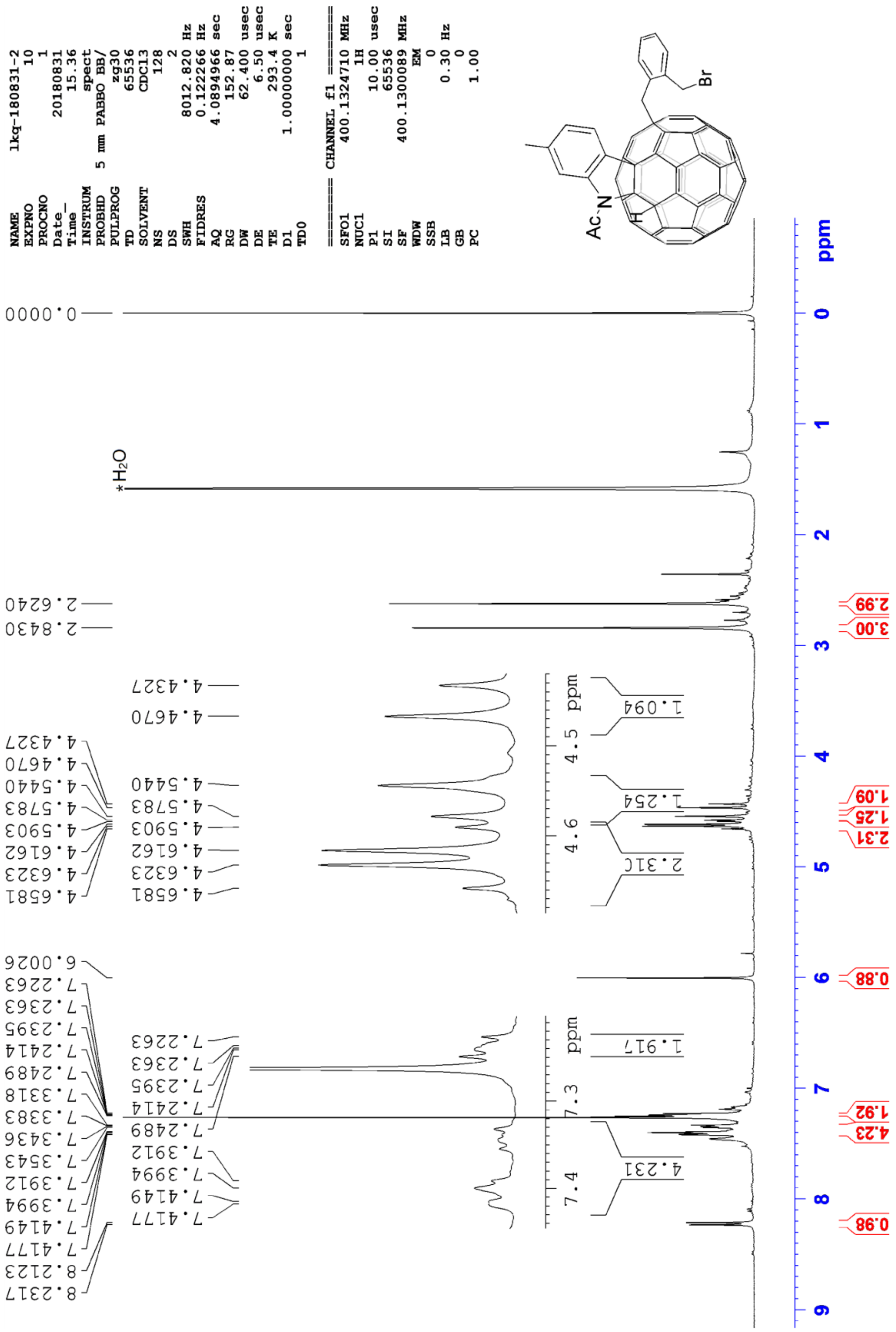

Figure S12. ^{1}H NMR (400 MHz, $CDCl_3$) spectrum of compound **3**.

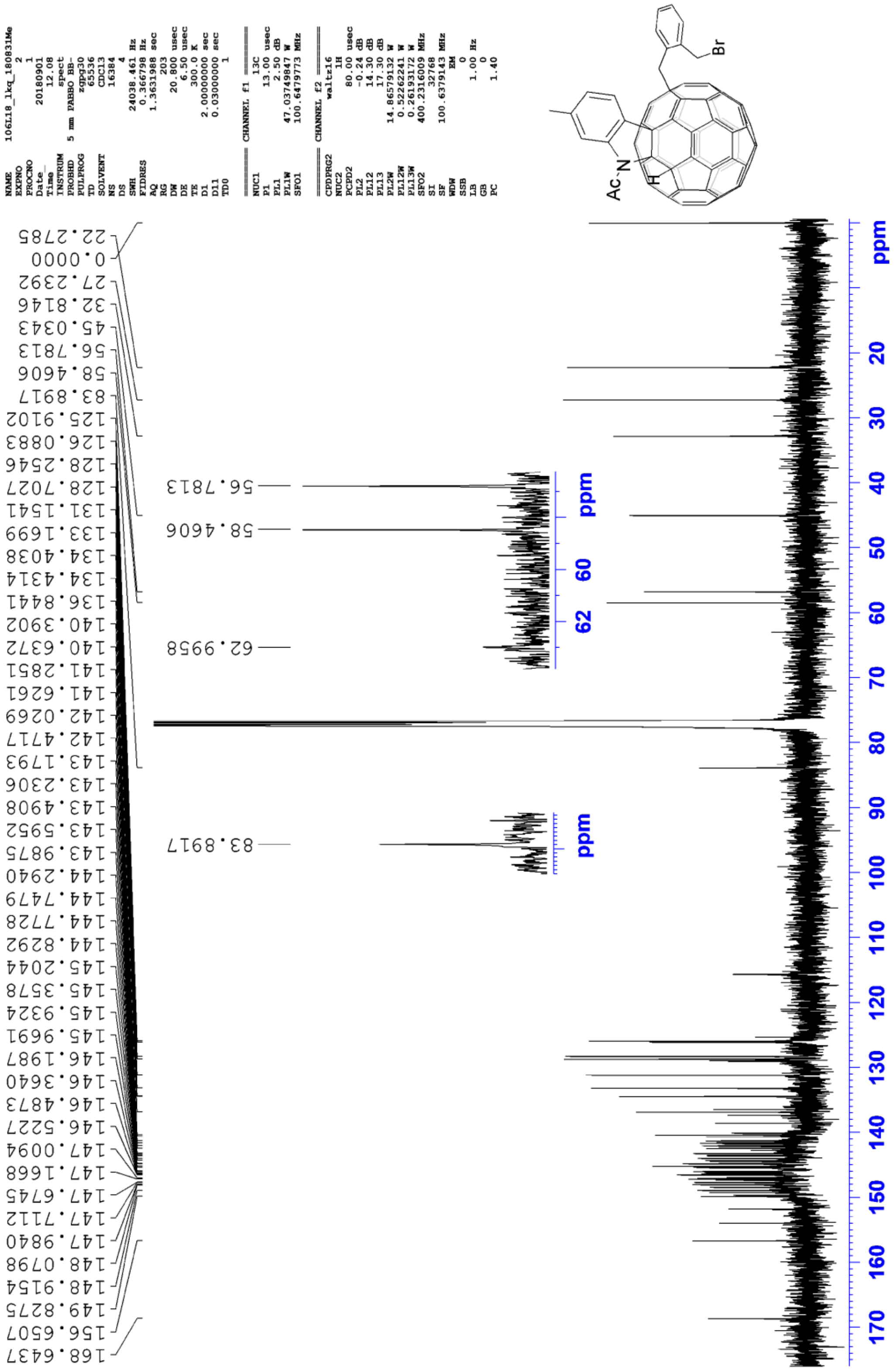

Figure S13. ^{13}C NMR (101 MHz, $CDCl_3$) spectrum of compound **3**.

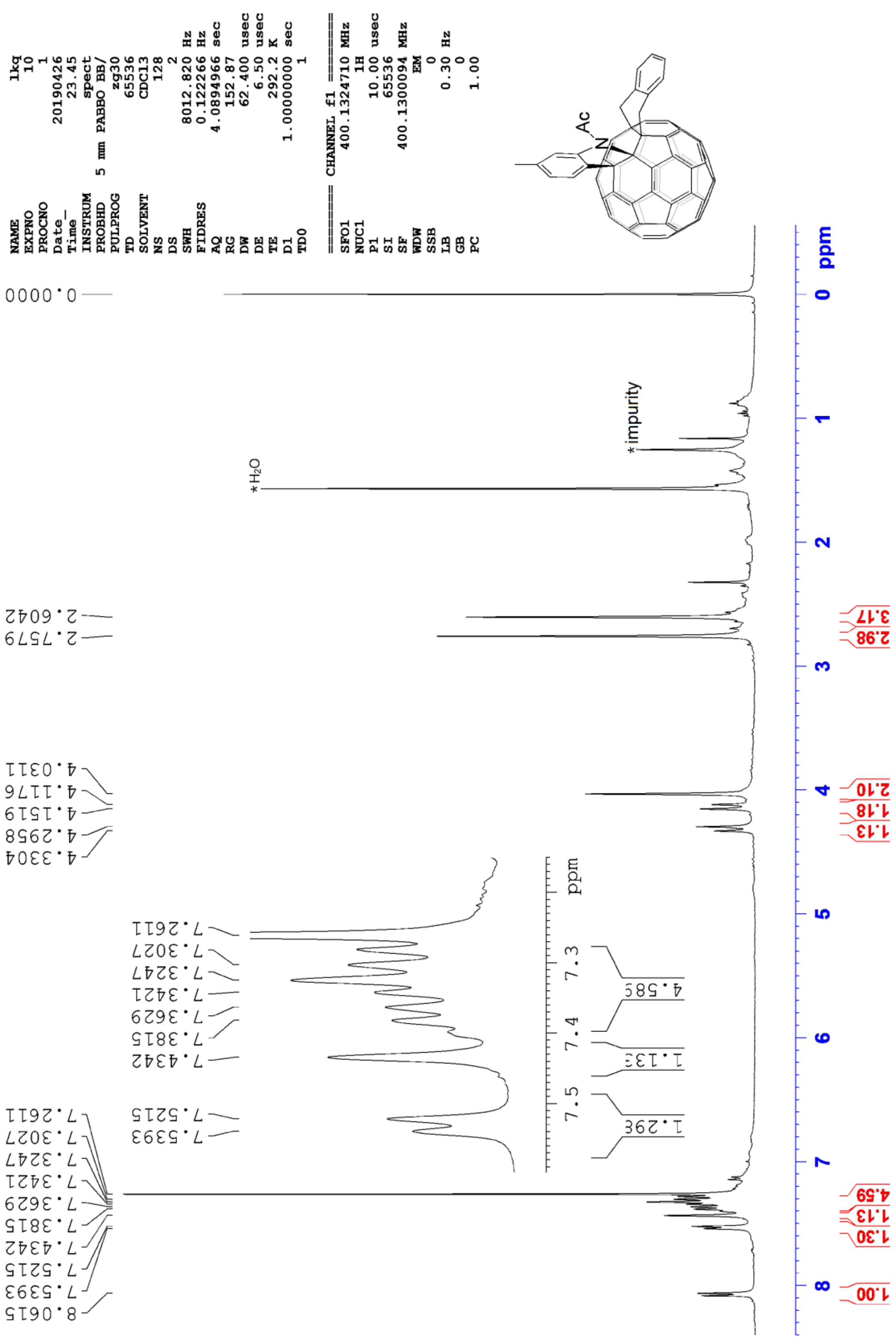

Figure S14. ^{1}H NMR (400 MHz, $CDCl_3$) spectrum of compound **4**.

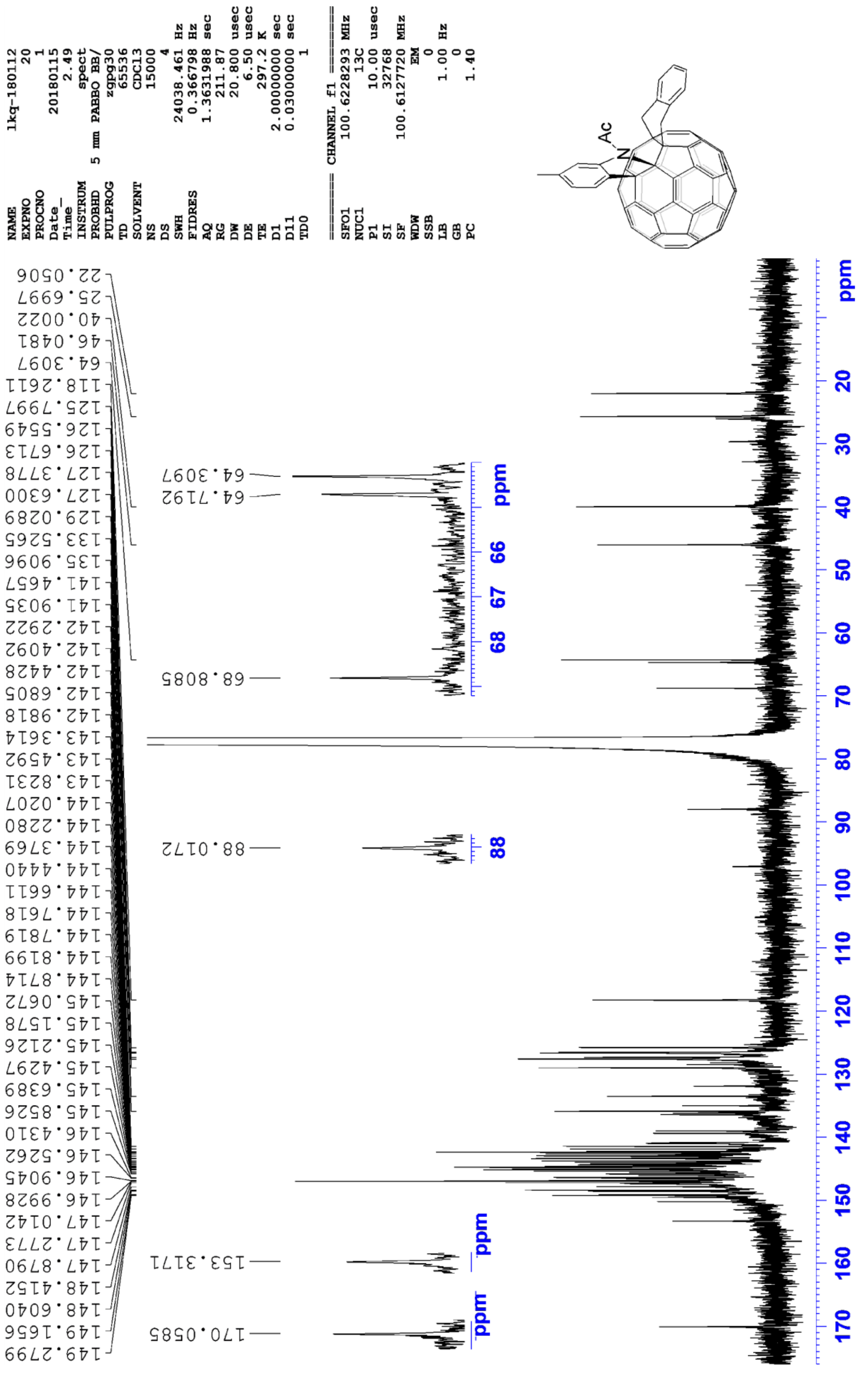

Figure S15. ^{13}C NMR (101 MHz, $CDCl_3$) spectrum of compound **4**.

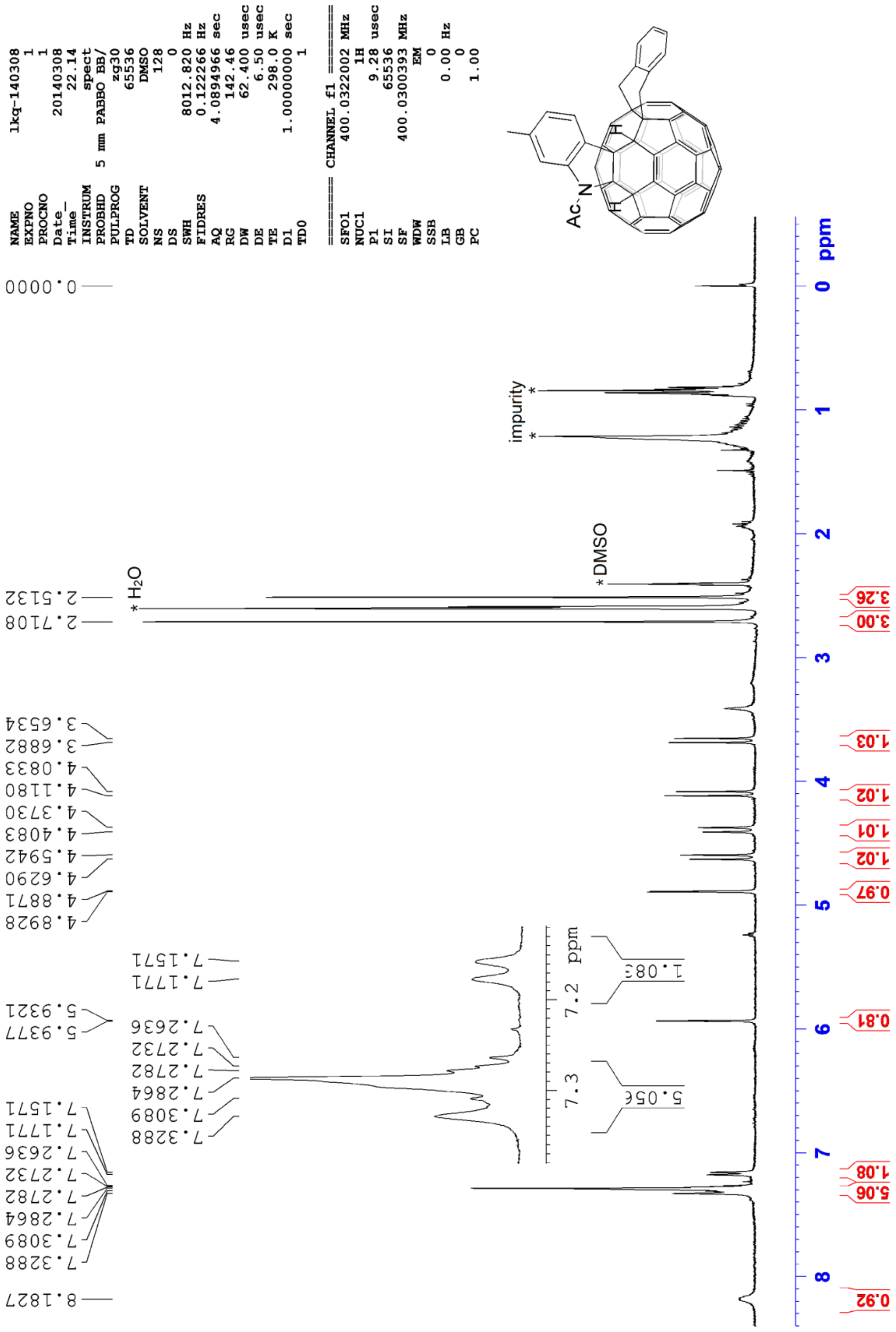

Figure S16. ^{1}H NMR (400 MHz, CS_2/DMSO-d_6) spectrum of compound **5**.

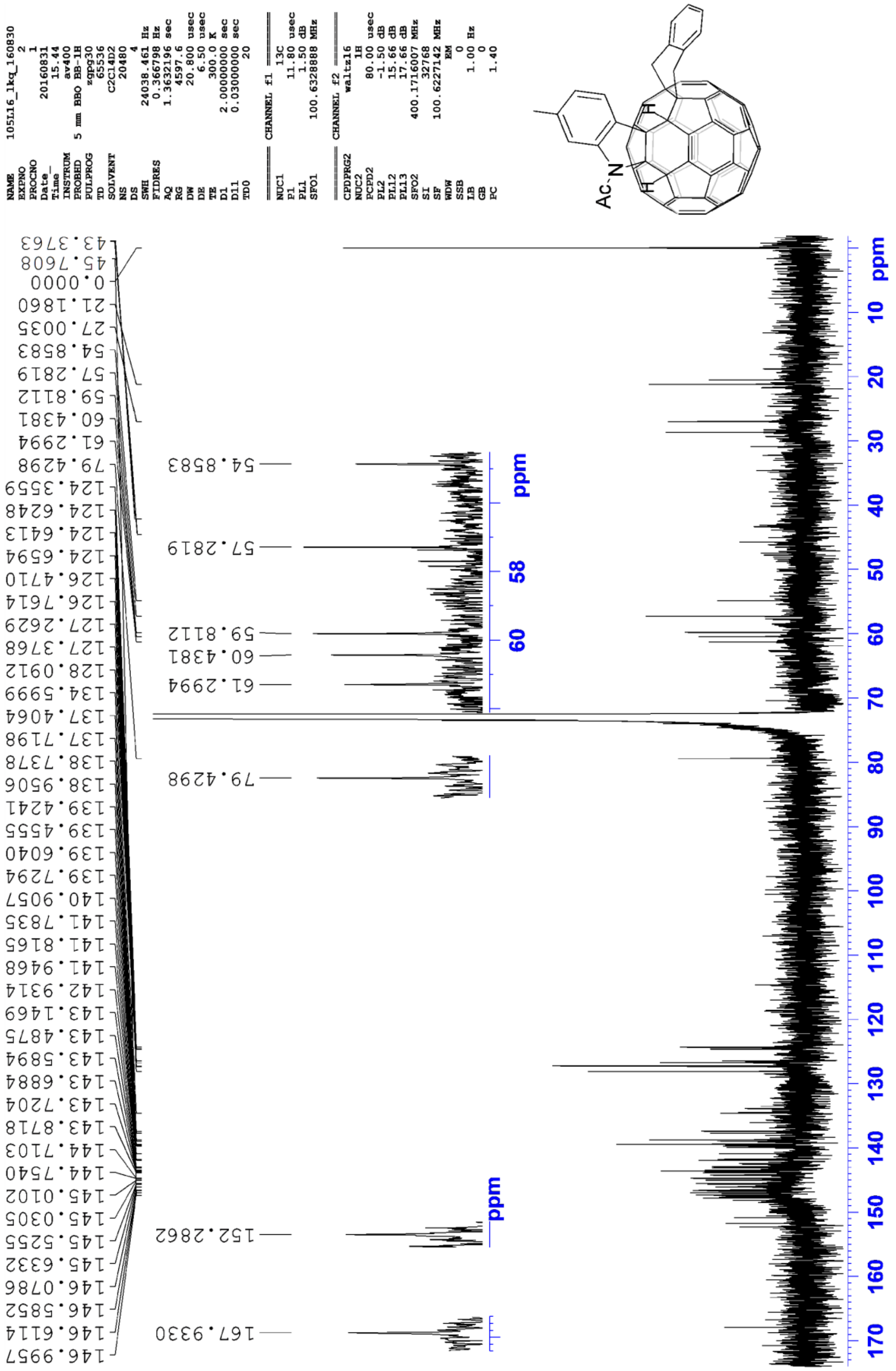

Figure S17. ^{13}C NMR (101 MHz, $C_2D_2Cl_4$) spectrum of compound **5**.

The xyz Coordinates and Energies for the Lowest Energy Structures of I–VII, 2, 2', 2'', and 4

The xyz coordinates for the lowest energy structure of **I**.

-1 1

C	-1.35187100	-0.47752200	-3.48210900
C	-0.90573900	0.87143000	-3.27097600
C	0.32320500	1.07459300	-2.59945000
C	1.17066700	-0.00257800	-2.22164400
C	0.72620300	-1.30675300	-2.38746900
C	-0.55292800	-1.54068200	-3.05084200
C	-2.78512100	-0.52613100	-3.26051300
C	-3.21790000	0.81432300	-2.89461400
C	-2.04573300	1.67305500	-2.90354000
C	-1.90412400	2.68065000	-1.94546500
C	0.47711400	2.14213800	-1.65710900
C	2.18457300	0.42142100	-1.17299000
C	2.42588600	-0.65111600	-0.02234700
C	1.88327900	-2.18366100	-0.27599400
C	0.99137000	-2.37637000	-1.46796400
C	-0.18594900	-3.16188800	-1.41423400
C	-1.13578600	-2.69122000	-2.42577500
C	-2.51483900	-2.75554300	-2.21520300
C	-3.35194800	-1.63776300	-2.63067800
C	-4.20143400	0.98709400	-1.91787700
C	-4.79161900	-0.16922000	-1.26935200
C	-4.37359700	-1.45682200	-1.61940700
C	-4.16213200	-2.46696400	-0.58852500
C	-3.03362100	-3.28434000	-0.97177600
C	-2.11348800	-3.71755900	0.00831500
C	-0.70048600	-3.72582100	-0.22206500
C	-0.04967300	-3.31035800	1.01399700
C	1.07832300	-2.49319500	0.99513300
C	1.72447500	1.93607000	-0.78701900
C	-0.22377900	0.08306900	3.30418400
C	0.33837300	1.17510800	2.64707600
C	-0.48822000	2.30204200	2.26749900
C	-1.86376500	2.27857100	2.53745700
C	-2.45572100	1.12086800	3.19107100
C	-2.09955800	-1.31788100	3.37528500
C	-0.94612600	-2.11556100	2.99155500
C	0.19944600	-1.24474300	2.94451000
C	1.14480600	-1.40461100	1.91680100
C	1.74201700	-0.26182400	1.27941300

C	1.35403300	1.00117600	1.63469100
C	0.03348800	2.84699000	1.04746300
C	-0.85044200	3.27126900	0.08297600
C	-2.27732600	3.22594600	0.31454700
C	-2.78409700	2.74597000	1.52903900
C	-3.95232900	1.88395300	1.54849200
C	-3.74424800	0.87334700	2.57459900
C	-4.17379600	-0.44292100	2.36536700
C	-3.33749400	-1.55581100	2.77017000
C	-1.07447200	-3.11622400	2.02439800
C	-2.35787900	-3.36741900	1.40775700
C	-3.47120400	-2.60607600	1.77000400
C	-4.40308800	-2.14617100	0.75431100
C	-4.82242000	-0.80573600	1.11393400
C	-5.02253200	0.16604800	0.12717300
C	-4.56666000	1.52550400	0.34411200
C	-4.05765100	2.03529800	-0.91924900
C	-2.92627100	2.85559400	-0.93303100
C	1.43404800	2.25414300	0.75858600
C	-0.61077900	2.92009000	-1.33032400
C	-1.65004900	0.04784200	3.57891600
C	2.84849100	2.88896700	-1.33467700
H	3.81888100	2.50094300	-0.99173500
H	2.86016500	2.82008100	-2.42796300
C	2.49580600	3.25738200	1.33353300
H	3.47687300	2.76304600	1.35305500
H	2.24010100	3.48090100	2.37424800
C	2.50210700	6.90582700	0.15767600
C	2.65022100	6.71431000	-1.21701000
C	2.75049700	5.42039200	-1.73442200
C	2.71383300	4.31662000	-0.88074100
C	2.56302900	4.50983100	0.50230400
C	2.45158300	5.80317100	1.01433400
H	2.41524600	7.91125700	0.56245600
H	2.67881800	7.56992600	-1.88736100
H	2.85554100	5.26726700	-2.80641500
H	2.31960300	5.94847100	2.08446700
C	3.91311300	-0.87150900	0.21366800
C	4.28713400	-2.21196400	0.02639500
C	4.86265400	0.05774600	0.61697800
C	5.60369000	-2.61253900	0.29547100
C	6.18633800	-0.33161500	0.84057000
H	4.57376800	1.09075400	0.78340200
C	6.56318600	-1.66882400	0.68807600

H	5.89981600	-3.65116200	0.23058700
H	6.92061500	0.40489600	1.15819400
N	3.18759600	-2.98586400	-0.36032600
C	3.16324800	-4.31848000	-0.74331100
O	2.13413600	-4.97540600	-0.70913000
C	4.43914500	-4.95392100	-1.28923000
H	5.10729700	-4.24109900	-1.77895600
H	4.99412600	-5.46592500	-0.49429800
H	4.11761900	-5.71367500	-2.00536600
C	7.99019500	-2.10565200	0.93450600
H	8.03183600	-3.08251200	1.43056100
H	8.55144400	-2.19799500	-0.00564300
H	8.52476300	-1.38598100	1.56379600
H	3.16111800	0.52793400	-1.66016100

E = -3074.90961482 a.u.

The xyz coordinates for the lowest energy structure of **II**.

-1 1

C	-1.26635200	-0.28142900	-3.55263300
C	-0.79279000	1.03375200	-3.20446800
C	0.41149400	1.19754100	-2.50928800
C	1.21346900	0.03903900	-2.07933000
C	0.77350200	-1.25229000	-2.48040500
C	-0.48223300	-1.38063200	-3.08897000
C	-2.68421400	-0.28944000	-3.32598300
C	-3.10045000	1.05476300	-2.92204800
C	-1.91433400	1.87774400	-2.86401100
C	-1.76366100	2.84939100	-1.85917300
C	0.56824400	2.21072700	-1.53622800
C	1.96594200	0.35154000	-0.96583600
C	2.38856800	-0.71034200	0.03881700
C	1.83548700	-2.22666600	-0.26675400
C	1.09915900	-2.48899300	-1.64842200
C	-0.28647400	-3.13548400	-1.55422300
C	-1.15465000	-2.55727900	-2.53925800
C	-2.54383500	-2.56707300	-2.38355700
C	-3.34553400	-1.40720100	-2.78897200
C	-4.11872700	1.21896500	-1.97610600
C	-4.78137300	0.06555400	-1.40610500
C	-4.37414100	-1.22554500	-1.80440000
C	-4.23635400	-2.27834900	-0.80277000
C	-3.10873600	-3.11157500	-1.17702500
C	-2.25582800	-3.62953200	-0.18892400
C	-0.82771800	-3.64413300	-0.39470100

C	-0.18842100	-3.31323600	0.89396700
C	0.94904800	-2.53152700	0.94218600
C	1.75340100	1.85457500	-0.62313800
C	-0.33632800	-0.03830400	3.32930900
C	0.28348800	1.05819400	2.73126600
C	-0.49927100	2.22393500	2.36661700
C	-1.88223400	2.23868000	2.59916000
C	-2.53141900	1.07367100	3.18082400
C	-2.25810000	-1.37397400	3.26884300
C	-1.11623300	-2.18740300	2.88414600
C	0.05911900	-1.35923900	2.92107900
C	1.05411300	-1.50817900	1.93611300
C	1.72984200	-0.36803200	1.38582800
C	1.33939400	0.89536100	1.76226800
C	0.07606500	2.79847800	1.18756700
C	-0.76031500	3.30665900	0.21573800
C	-2.19518800	3.29430800	0.40937400
C	-2.75293300	2.78230500	1.58580300
C	-3.94363200	1.95105900	1.52532200
C	-3.80805600	0.89799100	2.51926100
C	-4.27283000	-0.39286100	2.22994400
C	-3.48406600	-1.55031500	2.61558200
C	-1.24157100	-3.14023500	1.86858800
C	-2.51640800	-3.33188000	1.20560500
C	-3.61693600	-2.54854500	1.57068900
C	-4.49341800	-2.00678400	0.54301900
C	-4.90604000	-0.67721500	0.95655400
C	-5.02368000	0.33782900	-0.00512700
C	-4.53564800	1.67381300	0.29157800
C	-3.96890200	2.22104300	-0.93151900
C	-2.81748300	3.00896800	-0.87226900
C	1.46319000	2.17214100	0.91681800
C	-0.49288500	3.04234900	-1.20924900
C	-1.77384000	-0.03903500	3.55878900
C	2.98047400	2.70369600	-1.14597300
H	3.90347100	2.23877100	-0.78285200
H	3.00798800	2.62467600	-2.23707400
C	2.55537500	3.12417500	1.51962700
H	3.50188100	2.57012600	1.58869900
H	2.27288000	3.38239600	2.54571600
C	2.85608700	6.74962200	0.31522300
C	3.05297200	6.53576700	-1.05001000
C	3.08752400	5.23340300	-1.55442700
C	2.93943800	4.14005400	-0.69861500

C	2.73911500	4.35693100	0.67531100
C	2.69177800	5.65983400	1.17387600
H	2.81936300	7.76212100	0.71012600
H	3.17019400	7.38140700	-1.72362100
H	3.22805200	5.06420000	-2.61995000
H	2.52157800	5.82281900	2.23619800
C	3.89432000	-0.93430100	0.16609700
C	4.24764000	-2.27362200	-0.04122100
C	4.88869400	-0.00516600	0.44416500
C	5.59716200	-2.64642300	-0.09721100
C	6.23319600	-0.38317400	0.44837200
H	4.62344800	1.02100900	0.66949300
C	6.59966500	-1.69820900	0.14911400
H	5.89733700	-3.65521200	-0.34350800
H	7.00042300	0.35517800	0.66958100
N	3.09703600	-3.07130500	-0.21265600
C	2.98294800	-4.45166700	-0.17649900
O	1.91982400	-5.00317900	-0.43445500
C	4.17790900	-5.30286100	0.23116500
H	4.86501400	-5.45594300	-0.60874000
H	4.74191400	-4.87661100	1.06504900
H	3.77219300	-6.27538400	0.51557800
C	8.05572500	-2.09740400	0.05939200
H	8.21751200	-3.12212600	0.41352000
H	8.42201600	-2.05144000	-0.97558400
H	8.68820700	-1.43147400	0.65655500
H	1.76687500	-3.15975100	-2.20384600

E = -3074.88717978 a.u.

The xyz coordinates for the lowest energy structure of **III**.

-1 1

C	1.24286600	-0.42012800	3.75815000
C	0.76893800	0.94317200	3.23938300
C	-0.41432800	1.10826700	2.53979300
C	-1.22005600	-0.03854700	2.07175200
C	-0.71904200	-1.36169100	2.34797800
C	0.48953300	-1.54024900	3.06433600
C	2.71860100	-0.39316700	3.30162000
C	3.08478500	0.94043800	2.91198600
C	1.88402700	1.76278400	2.88855300
C	1.74374100	2.77684000	1.92414600
C	-0.58729200	2.15683700	1.59302600
C	-1.92042700	0.29876100	0.94931400
C	-2.36817600	-0.73151900	-0.08055100

C	-1.79115500	-2.24716500	0.18088700
C	-0.91271100	-2.39707400	1.38691200
C	0.28249700	-3.14970700	1.38110400
C	1.16378400	-2.62043000	2.43527800
C	2.55733900	-2.65987200	2.27028900
C	3.32924200	-1.47061500	2.68588900
C	4.10670800	1.17012100	1.97780500
C	4.76259600	0.04520200	1.36032800
C	4.36791300	-1.25359200	1.70335000
C	4.22726800	-2.27470100	0.68039800
C	3.11438200	-3.13972000	1.04479500
C	2.26452000	-3.64194600	0.02137800
C	0.84958600	-3.67875400	0.20831300
C	0.21067400	-3.30188200	-1.05299000
C	-0.95283200	-2.53821100	-1.07498900
C	-1.76494700	1.82196400	0.66711100
C	0.32363900	0.07907700	-3.35131400
C	-0.30146700	1.15237600	-2.71823700
C	0.47202600	2.31456900	-2.31904900
C	1.85520300	2.34481100	-2.54788300
C	2.51244200	1.20872300	-3.17815600
C	2.25718000	-1.24367800	-3.35482100
C	1.12282200	-2.08379900	-3.00166600
C	-0.06007400	-1.26095700	-2.99815200
C	-1.04559400	-1.45943400	-2.01130100
C	-1.71563900	-0.33633000	-1.41236600
C	-1.34708900	0.94400400	-1.75094400
C	-0.09837400	2.83602800	-1.11361200
C	0.73483900	3.32470900	-0.13159100
C	2.16798100	3.32818500	-0.32357100
C	2.72619600	2.85915000	-1.52074800
C	3.92448500	2.03839400	-1.49634800
C	3.78923100	1.01671800	-2.52138500
C	4.26050100	-0.28242800	-2.28198100
C	3.48083100	-1.43187600	-2.70436600
C	1.25987700	-3.07110600	-2.02491600
C	2.53438600	-3.27194800	-1.36102700
C	3.62954600	-2.47446300	-1.70210900
C	4.49977100	-1.96266900	-0.65374200
C	4.88565900	-0.60779200	-1.01415700
C	5.02380000	0.37812000	-0.03097100
C	4.52162300	1.71647000	-0.27327300
C	3.94842400	2.20834300	0.97024800
C	2.79348500	2.98936500	0.94348800

C	-1.48100300	2.19051600	-0.86187100
C	0.47306600	2.98117700	1.28177100
C	1.76111900	0.10040400	-3.57670500
C	-3.02480600	2.59821800	1.21109900
H	-3.92819100	2.12072600	0.81406100
H	-3.06306100	2.47069100	2.29736200
C	-2.57950200	3.15638600	-1.43005100
H	-3.51526500	2.59235700	-1.54301100
H	-2.28538300	3.47061700	-2.43679400
C	-2.98333000	6.71044400	-0.05447700
C	-3.19714800	6.42582200	1.29516400
C	-3.20715100	5.10004200	1.73533900
C	-3.01712700	4.05443800	0.82983100
C	-2.80013300	4.34176600	-0.52831200
C	-2.77799800	5.66838300	-0.96204300
H	-2.96597100	7.74149200	-0.39928800
H	-3.34711100	7.23438300	2.00655900
H	-3.36216200	4.87582100	2.78857300
H	-2.59619100	5.88692600	-2.01225900
C	-3.86840700	-0.96249300	-0.22593700
C	-4.20731700	-2.31348600	-0.03874100
C	-4.85016100	-0.05591200	-0.60146500
C	-5.52850300	-2.73561000	-0.24501000
C	-6.17582800	-0.46787700	-0.76480200
H	-4.58837600	0.97706900	-0.80024900
C	-6.52314700	-1.80930900	-0.58829300
H	-5.80311000	-3.77916900	-0.17226800
H	-6.93445900	0.25508600	-1.05504100
N	-3.07494600	-3.07774800	0.26461500
C	-3.00160300	-4.41881400	0.61217500
O	-1.95400100	-5.04118500	0.52879400
C	-4.23880800	-5.11002700	1.17739500
H	-4.89665800	-4.43799500	1.73435000
H	-4.82045900	-5.59025000	0.38163700
H	-3.87017000	-5.89892800	1.83675000
C	-7.95296900	-2.27082900	-0.76170600
H	-8.00267500	-3.25547400	-1.24135000
H	-8.46791100	-2.35729200	0.20511000
H	-8.52763800	-1.56829800	-1.37491600
H	1.17348900	-0.47136000	4.85957600

E = -3074.88624763 a.u.

The xyz coordinates for the lowest energy structure of **IV**.
-1 1

C	-1.31220900	-0.40943500	-3.43304900
C	-0.76020700	0.94029200	-3.20456300
C	0.44733400	1.09772000	-2.54342600
C	1.21631700	-0.06797300	-2.07531000
C	0.69335000	-1.38809500	-2.35097500
C	-0.54431900	-1.54090800	-3.05939600
C	-2.70681800	-0.34695200	-3.32708800
C	-3.05533300	0.99186700	-2.92256200
C	-1.84969200	1.81534700	-2.90477700
C	-1.67540700	2.82082200	-1.94568400
C	0.65698500	2.15781100	-1.61906100
C	1.93181900	0.26254700	-0.95919000
C	2.35490600	-0.77252800	0.07153600
C	1.73667000	-2.27621600	-0.18074700
C	0.83965000	-2.40626100	-1.38278800
C	-0.37475100	-3.14939700	-1.36331200
C	-1.24765300	-2.63574100	-2.40762900
C	-2.62364400	-2.62307900	-2.23926000
C	-3.53138600	-1.55378000	-2.88538000
C	-4.07281100	1.25601500	-1.99937600
C	-4.73581700	0.11625200	-1.34416300
C	-4.42476900	-1.18859500	-1.68210300
C	-4.26971300	-2.16674000	-0.63919800
C	-3.17110000	-3.04951400	-0.98900300
C	-2.33872600	-3.57554200	0.02434600
C	-0.92823800	-3.65656100	-0.17349900
C	-0.27536200	-3.28720600	1.07774700
C	0.90165700	-2.54034800	1.08398100
C	1.82291500	1.79292900	-0.68825500
C	-0.29962800	0.11837300	3.35244300
C	0.34208600	1.17587100	2.70575100
C	-0.40389200	2.34778600	2.30398800
C	-1.78498700	2.40944100	2.53175900
C	-2.46114500	1.29148600	3.17423700
C	-2.25793800	-1.16294100	3.37935400
C	-1.14539900	-2.02990300	3.02645500
C	0.05346000	-1.23341800	3.00712400
C	1.02215000	-1.45868700	2.00861700
C	1.71556000	-0.35118300	1.40114800
C	1.38198700	0.93714500	1.73424700
C	0.17506000	2.85118300	1.08673300
C	-0.64835500	3.34893000	0.10675200
C	-2.08656500	3.38381100	0.29674700
C	-2.64994400	2.93798500	1.50467700

C	-3.85334600	2.13326100	1.48426700
C	-3.74239900	1.11957300	2.52439100
C	-4.24858900	-0.16890200	2.30946700
C	-3.49335200	-1.33016200	2.74373100
C	-1.31317000	-3.02562000	2.05939000
C	-2.59559200	-3.20262600	1.41576600
C	-3.66619300	-2.37379600	1.74672400
C	-4.54123500	-1.85428100	0.70318300
C	-4.88709600	-0.49886200	1.04713800
C	-4.97722300	0.47427000	0.04143600
C	-4.44874100	1.79991400	0.26138500
C	-3.87773600	2.27509900	-0.99203300
C	-2.71739800	3.06588800	-0.96306200
C	1.54414500	2.17549000	0.83977700
C	-0.39385400	2.99685800	-1.30760900
C	-1.73124800	0.17254200	3.58840600
C	3.10929800	2.52457900	-1.22873200
H	3.99690600	2.02586100	-0.82195700
H	3.15046000	2.38732500	-2.31369600
C	2.66414700	3.11806400	1.40440500
H	3.58498100	2.53158500	1.52755100
H	2.37260000	3.44839800	2.40671900
C	3.16962400	6.64760400	-0.00034700
C	3.38528800	6.34491900	-1.34575300
C	3.36318400	5.01532900	-1.77377500
C	3.13917100	3.98390100	-0.85995400
C	2.92071400	4.28924400	0.49389000
C	2.93052800	5.61992000	0.91529600
H	3.17656800	7.68194600	0.33479200
H	3.56095800	7.14259100	-2.06348700
H	3.51866100	4.77740900	-2.82389100
H	2.74686300	5.85296400	1.96203100
C	3.84741700	-1.04685500	0.21423100
C	4.14752400	-2.40530500	0.02271500
C	4.85321200	-0.16984500	0.59705400
C	5.45426400	-2.86754800	0.23230000
C	6.16546100	-0.62125600	0.76149300
H	4.62023000	0.86884400	0.80168200
C	6.47458700	-1.97163400	0.58078100
H	5.69787500	-3.91848100	0.15741900
H	6.94401400	0.07805400	1.05662200
N	2.99305000	-3.13636000	-0.28807300
C	2.88391300	-4.47168400	-0.64972800
O	1.81733000	-5.06282100	-0.58161300

C	4.10500200	-5.19295600	-1.21062800
H	4.78432300	-4.53631400	-1.76001300
H	4.66821600	-5.69239000	-0.41357300
H	3.71926400	-5.96837600	-1.87603000
C	7.89008600	-2.47457200	0.75553000
H	7.91020200	-3.46502000	1.22509400
H	8.40644400	-2.56520100	-0.21009600
H	8.48127500	-1.79454900	1.37822700
H	-4.12876200	-1.97651200	-3.71189500

E = -3074.88083570 a.u.

The xyz coordinates for the lowest energy structure of **V**.

0 1

C	-1.37704600	-0.37742600	-3.48489000
C	-0.92199200	0.96057000	-3.22205800
C	0.30305500	1.14632400	-2.56328600
C	1.13635200	0.03582100	-2.20538500
C	0.70933200	-1.23519900	-2.46434100
C	-0.57612500	-1.44535400	-3.08232300
C	-2.81220000	-0.41765800	-3.26507300
C	-3.23123900	0.91635300	-2.86533700
C	-2.05509000	1.76709500	-2.85528500
C	-1.89421700	2.73907600	-1.86197500
C	0.48040100	2.18339100	-1.60285500
C	2.17748200	0.43727300	-1.18085900
C	2.43366100	-0.66124900	-0.06478900
C	1.90451500	-2.19209700	-0.34193600
C	1.12231700	-2.47667000	-1.68797700
C	-0.24581700	-3.16602000	-1.54397800
C	-1.17176400	-2.62949200	-2.50160000
C	-2.55834700	-2.67718200	-2.29745200
C	-3.38851000	-1.54097600	-2.67216600
C	-4.20999000	1.07268800	-1.88012200
C	-4.80741200	-0.09535600	-1.26260100
C	-4.40731500	-1.37836600	-1.65325600
C	-4.20550000	-2.41314100	-0.65240900
C	-3.06523300	-3.21624100	-1.06057700
C	-2.15929900	-3.68386000	-0.10290600
C	-0.73760000	-3.66228900	-0.35937600
C	-0.06218300	-3.31096900	0.90272100
C	1.05149000	-2.49478300	0.89337800
C	1.73444800	1.94953100	-0.75466200
C	-0.20648700	-0.00018400	3.29389400
C	0.35763600	1.10276300	2.65689000

C	-0.46332400	2.24598000	2.31929400
C	-1.83695800	2.22303600	2.59574600
C	-2.43265900	1.05101000	3.21962000
C	-2.09160900	-1.39177600	3.32693500
C	-0.94500100	-2.17995700	2.91119900
C	0.20436400	-1.31623000	2.88595300
C	1.14956400	-1.44799900	1.85672500
C	1.75653700	-0.29724300	1.25030700
C	1.37267500	0.95706700	1.64040700
C	0.05339700	2.81499700	1.10786400
C	-0.83093900	3.28592000	0.16760600
C	-2.25666100	3.23398300	0.40577000
C	-2.75946100	2.72688300	1.60855900
C	-3.92761100	1.86320400	1.60221200
C	-3.72576000	0.82779800	2.60327400
C	-4.16635700	-0.47746100	2.35840700
C	-3.33522500	-1.60683300	2.72717000
C	-1.08106100	-3.15041100	1.91351000
C	-2.37550800	-3.37903100	1.30179800
C	-3.48031500	-2.62483200	1.69831600
C	-4.41075500	-2.12466500	0.69817200
C	-4.82892000	-0.79669500	1.10459300
C	-5.02295000	0.20030400	0.14403800
C	-4.56060900	1.55204100	0.39726700
C	-4.04972600	2.09024900	-0.85397100
C	-2.91599200	2.90552600	-0.84689800
C	1.45356100	2.23161500	0.79929900
C	-0.60213700	2.96315200	-1.25375100
C	-1.63178600	-0.03536100	3.57250000
C	2.85521500	2.91332400	-1.29019100
H	3.82773600	2.51753200	-0.96554600
H	2.85821800	2.87043500	-2.38483000
C	2.51724900	3.22311900	1.39269000
H	3.49639000	2.72568700	1.40006700
H	2.26546900	3.42458200	2.43862200
C	2.49939400	6.89267600	0.29313100
C	2.63842000	6.73161900	-1.08643200
C	2.74168900	5.45043900	-1.63340400
C	2.71694600	4.32970300	-0.80185000
C	2.57518100	4.49215800	0.58536000
C	2.46050300	5.77281300	1.12738200
H	2.41314800	7.88841300	0.71959400
H	2.66022400	7.60149200	-1.73730100
H	2.84357500	5.32259500	-2.70874300

H	2.33916900	5.89608600	2.20120500
C	3.92557300	-0.87585500	0.14024400
C	4.30368300	-2.21333900	-0.02098700
C	4.88041000	0.08336800	0.45676100
C	5.65335900	-2.57187600	0.09672400
C	6.22126200	-0.27482400	0.59124200
H	4.58343100	1.11488200	0.61378700
C	6.61842000	-1.60191700	0.40097600
H	5.99041700	-3.58664200	-0.04594900
H	6.96020700	0.48076600	0.84398000
N	3.17511300	-3.01533200	-0.31108100
C	3.06535700	-4.39905700	-0.39905400
O	1.97424300	-4.91528100	-0.60525300
C	4.29132100	-5.27909700	-0.24366600
H	5.02151300	-5.10809700	-1.04137800
H	4.78796100	-5.12612600	0.71943000
H	3.93812800	-6.30905800	-0.30527000
C	8.07556900	-1.99379600	0.49353700
H	8.19561600	-3.01861700	0.86059700
H	8.56409900	-1.94030500	-0.48855900
H	8.62532900	-1.32751300	1.16611900
H	3.13730400	0.54746300	-1.69641100
H	1.77842300	-3.10461200	-2.30059300

E = -3075.45158534 a.u.

The xyz coordinates for the lowest energy structure of **VI**.

0 1

C	-1.31906900	-0.46964400	-3.49420000
C	-0.88011700	0.88466600	-3.26094400
C	0.34115500	1.10618500	-2.59803200
C	1.17743800	0.01291100	-2.19552600
C	0.75253800	-1.27442500	-2.40652500
C	-0.51059700	-1.52261600	-3.06448900
C	-2.74662300	-0.52436200	-3.28008200
C	-3.19100500	0.81287300	-2.91723500
C	-2.02695600	1.68428000	-2.92074500
C	-1.88829500	2.67851000	-1.94851000
C	0.49177500	2.16297700	-1.65226700
C	2.18625300	0.43940900	-1.14688400
C	2.42784000	-0.62967400	0.00631700
C	1.89582300	-2.16254400	-0.24031700
C	0.95749700	-2.32936000	-1.41072800
C	-0.14725800	-3.13817700	-1.40420600
C	-1.09006300	-2.67436200	-2.42293800

C	-2.45909700	-2.74266400	-2.22687200
C	-3.30318500	-1.64366200	-2.64367400
C	-4.17502500	0.97749800	-1.94239400
C	-4.76196800	-0.18029200	-1.29422400
C	-4.32951100	-1.46435000	-1.63380300
C	-4.13064600	-2.46208100	-0.59504500
C	-2.98259000	-3.26558600	-0.94742100
C	-2.11829200	-3.72460800	0.02733500
C	-0.61157800	-3.94123000	-0.21188100
C	-0.01809900	-3.31116200	1.05262400
C	1.08067800	-2.47670100	1.02733700
C	1.73140400	1.95704400	-0.77195000
C	-0.24576200	0.10526100	3.31319000
C	0.31394800	1.19477900	2.64838400
C	-0.51164700	2.31694200	2.26837300
C	-1.88793800	2.29042100	2.52967100
C	-2.48032300	1.13431400	3.18202400
C	-2.11524800	-1.30179600	3.36309500
C	-0.95459700	-2.09174900	2.98868600
C	0.18417400	-1.21874800	2.95142000
C	1.14252600	-1.37826700	1.94067400
C	1.73666300	-0.23701300	1.30094000
C	1.34169700	1.02558500	1.64758200
C	0.01545100	2.85407100	1.04249100
C	-0.85870200	3.28862300	0.07941000
C	-2.28923400	3.22957400	0.30206500
C	-2.80288900	2.75468100	1.51452100
C	-3.96019800	1.88030400	1.52138700
C	-3.76324400	0.87914500	2.55717100
C	-4.18941100	-0.43761700	2.34940700
C	-3.35041000	-1.54788600	2.76009100
C	-1.06264700	-3.09205400	2.01471300
C	-2.34889100	-3.35356200	1.39427100
C	-3.47359300	-2.59817100	1.76296700
C	-4.38536900	-2.14164000	0.74236000
C	-4.82742400	-0.80396400	1.09637600
C	-5.00975900	0.15747600	0.09984200
C	-4.56926400	1.52124400	0.31476200
C	-4.04724300	2.02855400	-0.94270900
C	-2.92610800	2.85953600	-0.94528200
C	1.42373700	2.27617200	0.77021700
C	-0.60769200	2.93226800	-1.33081500
C	-1.67251600	0.06518500	3.57481300
C	2.86425600	2.90628700	-1.30708800

H	3.82725200	2.52054500	-0.94254600
H	2.89693200	2.82931200	-2.39936300
C	2.47007700	3.29248900	1.35338700
H	3.45211300	2.80182900	1.39080800
H	2.19901800	3.52121300	2.38884000
C	2.45510000	6.92856800	0.14778100
C	2.62605700	6.72791000	-1.22303700
C	2.74813900	5.43191700	-1.72982500
C	2.71007900	4.33612800	-0.86619400
C	2.53641400	4.53839300	0.51204000
C	2.40325500	5.83357600	1.01376300
H	2.35442700	7.93594300	0.54258000
H	2.65838800	7.57853300	-1.89845100
H	2.87536900	5.27316300	-2.79832300
H	2.25770400	5.98764400	2.08057400
C	3.91446900	-0.85689500	0.23620200
C	4.28474400	-2.18866500	0.01664600
C	4.86405200	0.06461000	0.66097700
C	5.60051700	-2.60027000	0.26050100
C	6.18566300	-0.33383800	0.86520600
H	4.57716900	1.09270000	0.85863900
C	6.56269300	-1.66740000	0.67167000
H	5.89897200	-3.63439700	0.16068600
H	6.92231400	0.39177200	1.19949800
N	3.16863200	-2.95709300	-0.37808600
C	3.12209600	-4.29819100	-0.73605600
O	2.07072800	-4.92259100	-0.69897700
C	4.38358200	-4.97425200	-1.24714900
H	5.05908800	-4.29383200	-1.77060200
H	4.93026800	-5.45215700	-0.42626700
H	4.05478700	-5.76488100	-1.92472300
C	7.99195500	-2.10707100	0.89258100
H	8.04330600	-3.13513700	1.26701600
H	8.56674800	-2.07258400	-0.04251000
H	8.50221500	-1.45912000	1.61238700
H	3.15732200	0.53764900	-1.64411900
H	-0.34161400	-5.00071700	-0.31305300

E = -3075.44150013 a.u.

The xyz coordinates for the lowest energy structure of **VII**.

0 1

C	-1.34055200	-0.43854700	-3.48796600
C	-0.87626200	0.90705000	-3.25926400
C	0.34893600	1.09928000	-2.59304700

C	1.17050900	-0.00640300	-2.19846200
C	0.72020500	-1.29006900	-2.40749700
C	-0.55409600	-1.50926200	-3.06243200
C	-2.76458200	-0.46805500	-3.26331900
C	-3.18453200	0.87740400	-2.89655400
C	-2.00649300	1.72487900	-2.90691800
C	-1.84547800	2.71706900	-1.93360700
C	0.52309700	2.15058500	-1.64612100
C	2.19343000	0.40344400	-1.15639900
C	2.42576700	-0.66942000	-0.00713200
C	1.85791000	-2.18853900	-0.25258800
C	0.92876400	-2.35482100	-1.43654600
C	-0.20691700	-3.14451000	-1.42037700
C	-1.15673600	-2.65397900	-2.43718000
C	-2.52097000	-2.70691900	-2.24370300
C	-3.33056600	-1.57940400	-2.62047700
C	-4.16146200	1.05802400	-1.91712400
C	-4.76636600	-0.08715800	-1.26775400
C	-4.34973000	-1.38661200	-1.60386200
C	-4.19047300	-2.39335900	-0.59399400
C	-3.15343000	-3.43744400	-1.04932200
C	-2.11515200	-3.68928400	0.03201800
C	-0.75263800	-3.66260800	-0.19219900
C	-0.06884500	-3.28409600	1.05884700
C	1.05383600	-2.48039500	1.02756600
C	1.76584700	1.92599300	-0.77661400
C	-0.20550600	0.10830800	3.32739700
C	0.36493000	1.18579400	2.65459500
C	-0.44755000	2.32339900	2.27437400
C	-1.82257500	2.31533400	2.54255700
C	-2.42669500	1.16675700	3.19794200
C	-2.09535800	-1.27250500	3.39541900
C	-0.95063500	-2.08326300	3.02215800
C	0.20384800	-1.22377700	2.97009600
C	1.15647300	-1.39679000	1.95211900
C	1.75967300	-0.26639200	1.30023200
C	1.38231000	1.00181100	1.64780100
C	0.07843400	2.85027700	1.04986100
C	-0.79514500	3.30494400	0.09027500
C	-2.22128400	3.27256100	0.31941200
C	-2.73601200	2.79630900	1.53448800
C	-3.90543100	1.94372700	1.54327600
C	-3.71261200	0.93077900	2.57388300
C	-4.14607000	-0.37932600	2.35364800

C	-3.32867900	-1.50225300	2.77080500
C	-1.08628800	-3.08663200	2.06119400
C	-2.36659100	-3.32205900	1.42465300
C	-3.46381900	-2.54961600	1.77727300
C	-4.38765400	-2.06365100	0.73256400
C	-4.79681800	-0.72819100	1.10142300
C	-4.99447300	0.24736000	0.11906500
C	-4.53199300	1.60462800	0.34128100
C	-4.01070900	2.10731200	-0.91755700
C	-2.87340500	2.91644100	-0.92501300
C	1.47531900	2.24960200	0.76732200
C	-0.55940600	2.94438600	-1.32027200
C	-1.63429900	0.08732400	3.59487600
C	2.90694900	2.85980900	-1.32256700
H	3.86739300	2.45967000	-0.96736000
H	2.92817400	2.78298900	-2.41515300
C	2.54297000	3.24901600	1.34160100
H	3.51719900	2.74235500	1.36878400
H	2.28543500	3.48064000	2.37979700
C	2.57073700	6.88611700	0.13918800
C	2.72567200	6.68425700	-1.23335700
C	2.82393800	5.38710700	-1.74234900
C	2.77799900	4.29127900	-0.87916700
C	2.62049400	4.49468700	0.50083500
C	2.51102600	5.79122600	1.00481200
H	2.48875100	7.89450800	0.53569700
H	2.76416000	7.53495100	-1.90835300
H	2.93879000	5.22750000	-2.81212300
H	2.37792800	5.94638400	2.07307700
C	3.91008400	-0.92722700	0.20616400
C	4.24778100	-2.27010000	-0.00093100
C	4.88447800	-0.02085700	0.60636200
C	5.55909800	-2.70518400	0.22784100
C	6.20014400	-0.44440600	0.79796400
H	4.62214400	1.01571400	0.79432800
C	6.54594300	-1.78781500	0.61462500
H	5.83584500	-3.74557700	0.13300900
H	6.95658600	0.26935300	1.11300400
N	3.11136900	-3.01735200	-0.37328200
C	3.01948400	-4.36931900	-0.69611200
O	1.94695600	-4.95133800	-0.64527400
C	4.26019800	-5.10046500	-1.18205400
H	4.94612700	-4.46277800	-1.74485500
H	4.80412500	-5.54872400	-0.34277000

H	3.90656200	-5.91565700	-1.81644000
C	7.96904100	-2.25480200	0.81910000
H	8.00482800	-3.28110500	1.20006500
H	8.53152000	-2.23829700	-0.12395700
H	8.50222000	-1.61239700	1.52723000
H	3.16276100	0.48519100	-1.66012100
H	-3.64199100	-4.37514000	-1.36112200

E = -3075.42172403 a.u.

The xyz coordinates for the lowest energy structure of **2**

0 1

C	-5.67647085	-0.59723241	8.92841271
C	-6.90865766	-0.62978746	8.15399477
C	-7.02245654	-1.49750666	7.05025172
C	-5.90322088	-2.38906681	6.72254017
C	-4.73108455	-2.37006365	7.48849913
C	-4.61062981	-1.45911717	8.62195083
C	-5.40812031	0.79131014	9.29883635
C	-6.47367772	1.61961665	8.73689993
C	-7.39152351	0.73852821	8.02193284
C	-7.95862791	1.16685057	6.81591664
C	-7.64724194	-1.07433567	5.81758379
C	-5.82535696	-2.47744774	5.27470834
C	-4.58125132	-2.56813661	4.63725013
C	-3.35711935	-2.54810734	5.43080629
C	-3.42939230	-2.44646693	6.82651115
C	-2.50811802	-1.57223948	7.54270551
C	-3.24234150	-0.95458811	8.64399447
C	-2.98509146	0.37979950	8.99152005
C	-4.09427048	1.27063195	9.32386691
C	-6.17908573	2.88909426	8.22771085
C	-4.80456884	3.38898685	8.25220817
C	-3.78651128	2.59764198	8.79334042
C	-2.48917488	2.52049346	8.12335640
C	-1.99542901	1.14955032	8.24912139
C	-1.28034970	0.55703590	7.19978290
C	-1.54955191	-0.83159424	6.83496461
C	-1.47855405	-0.93424225	5.38250354
C	-2.36411086	-1.77132756	4.69509218
C	-6.88581560	-1.65450137	4.70978466
C	-3.74984617	0.76645304	2.34646108
C	-5.05440289	0.30569426	2.32972481
C	-6.30083175	1.23120536	2.34986210
C	-5.87421378	2.52131268	3.02943247

C	-4.57158448	3.00416679	3.04498778
C	-2.23999163	2.18711275	3.52417828
C	-1.78495031	0.83119890	3.66607077
C	-2.71331873	-0.04000362	2.95369919
C	-2.98819455	-1.31758890	3.45509275
C	-4.34930713	-1.80604763	3.42811183
C	-5.37203991	-0.97643964	2.91631847
C	-7.41656452	0.35227211	3.18215609
C	-7.91298086	1.00157034	4.45466837
C	-7.57045570	2.33689154	4.81585559
C	-6.63549871	3.12597354	4.11389193
C	-5.73265159	3.98870734	4.84178254
C	-4.43152814	3.90725926	4.18029387
C	-3.25931170	3.97186310	4.93739980
C	-2.14272977	3.05583304	4.59887913
C	-1.17201892	0.39269841	4.85322209
C	-1.04719923	1.30836067	5.97043317
C	-1.53581990	2.61808358	5.84410925
C	-2.26865585	3.23762606	6.94341588
C	-3.32346339	4.08104246	6.37715511
C	-4.57831938	4.14854097	7.02246957
C	-5.79392223	4.11439847	6.24099826
C	-6.78401506	3.32821515	6.97454023
C	-7.64898456	2.47625481	6.28692931
C	-6.67265256	-0.94845877	3.52724665
C	-8.07778627	0.25651257	5.67236320
C	-3.36827824	2.26610723	2.46092661
C	-6.96537532	1.42933322	0.99302226
C	-6.48507880	2.15548252	-0.09053199
C	-8.19455504	0.74879855	0.89697101
C	-7.19209388	2.17183239	-1.30141147
H	-5.56340466	2.71654906	0.00120412
C	-8.88678682	0.73510612	-0.32577784
C	-8.38758567	1.45029434	-1.43099747
H	-6.80761253	2.73886659	-2.14466585
H	-9.80203515	0.17842383	-0.45551961
N	-8.52448817	0.13539115	2.12822158
C	-9.67342143	-0.54868971	2.48985901
O	-9.72991586	-1.17378966	3.57350638
C	-10.90086323	-0.52326407	1.59840673
H	-11.12788026	0.47935107	1.22378333
H	-10.79365903	-1.20100829	0.74305512
H	-11.73143435	-0.88022109	2.21032188
C	-9.16103946	1.45252745	-2.73443339

H	-9.52489238	0.44875385	-2.98670640
H	-10.03771848	2.11296145	-2.67849199
H	-8.53694106	1.80242309	-3.56383972
C	-2.86444600	2.94104400	1.12215200
H	-1.77800927	2.82066536	1.09875534
H	-3.26105105	2.33979153	0.29824139
C	-3.19188915	4.40819749	0.93343577
C	-2.29647254	5.31835510	0.25732741
C	-4.39510472	4.90425504	1.45131010
C	-2.69731103	6.69199208	0.15033214
C	-4.76303724	6.25227809	1.32721824
C	-3.90122754	7.14880501	0.66865315
H	-2.02862199	7.38056999	-0.36010658
H	-5.70819123	6.59713373	1.73558183
H	-4.17699269	8.19443576	0.56441458
H	-1.36655593	4.97983269	-0.14956038
C	-5.34058750	3.92433183	2.17761781
H	-5.88273349	3.35449193	1.45217987
H	-6.03098927	4.47977114	2.77739491

E = -3074.20889121 a.u.

The xyz coordinates for the lowest energy structure of **2'**

0 1

C	4.13377400	-2.39970500	-0.72619800
C	3.00711000	-3.32587800	-0.68403500
C	2.01553600	-3.22119000	-1.63046900
C	2.12507600	-2.23047400	-2.69346900
C	3.21094300	-1.38809300	-2.75073000
C	4.24806500	-1.47838900	-1.73148000
C	4.46582900	-2.03810400	0.64516700
C	3.52076400	-2.71707700	1.51743100
C	2.63822400	-3.52545800	0.70039300
C	1.30707400	-3.63840900	1.04315400
C	0.62500500	-3.36125600	-1.26013700
C	0.80811900	-1.70166100	-2.94407000
C	0.63185700	-0.40503300	-3.29734500
C	1.76238900	0.47971700	-3.37033500
C	3.92464300	0.79343200	-2.27658900
C	4.66822000	-0.11773500	-1.42203300
C	4.97885900	0.23326400	-0.13386900
C	4.85007000	-0.75362700	0.92933300
C	3.03656800	-2.08152400	2.63663500
C	3.45925200	-0.72263500	2.93565800
C	4.35298100	-0.07932600	2.11694300

C	4.15716500	1.32502700	1.79119800
C	4.52478500	1.50869800	0.39163900
C	3.81661800	2.37190100	-0.40326100
C	3.49810000	2.00326700	-1.77105200
C	2.17135300	2.49672300	-2.07773300
C	1.32763100	1.76342900	-2.84897500
C	-0.15213000	-2.39667100	-2.06250300
C	-1.81623200	1.79995900	-0.49362300
C	-1.95811000	0.29775200	-0.74845000
C	-2.38292000	-0.71905900	0.35225600
C	-1.69742800	-0.34091300	1.62630700
C	-1.25083500	1.04161200	1.88121000
C	-0.02834900	2.83913300	0.84335600
C	0.33699100	3.04567100	-0.51663400
C	-0.75402700	2.55570400	-1.47876400
C	-0.09583300	1.63023300	-2.47133500
C	-0.48333600	0.36510400	-2.69683100
C	-1.40352700	-0.31911500	-1.79646300
C	-1.90269100	-2.14576200	-0.25678100
C	-0.89867200	-2.88446900	0.61820000
C	-0.57202900	-2.48066900	1.86646600
C	-0.99329300	-1.19377800	2.39411800
C	-0.05062800	-0.41837200	3.23567100
C	-0.24073000	0.97477600	2.91454200
C	0.83060400	1.83550000	2.90431100
C	0.94258800	2.79498400	1.82941200
C	1.64751500	3.16460700	-0.85881400
C	2.66793500	3.07638900	0.15266800
C	2.33266800	2.92929000	1.47646100
C	3.09358400	2.01087000	2.31292700
C	2.15581500	1.33892900	3.20291800
C	2.32982100	0.00428400	3.49181600
C	1.19208600	-0.88704000	3.51462800
C	1.63131000	-2.19587900	2.96839600
C	0.80007700	-2.95210200	2.20792400
C	-1.20225600	-1.74983600	-1.55324000
C	0.27515100	-3.57446400	0.03480100
C	-1.16342500	1.95931600	0.88900400
C	-3.87064800	-0.92210500	0.54881400
C	-4.74534400	-0.11101300	1.23385900
C	-4.30254400	-2.12154800	-0.00169800
C	-6.09169800	-0.44594300	1.29428500
H	-4.39332800	0.76877700	1.73706700
C	-5.64584700	-2.45818800	0.05201300

C	-6.54743800	-1.60742100	0.69185500
H	-6.77738500	0.19008100	1.82064600
H	-6.02052200	-3.36519600	-0.36299800
N	-3.20022100	-2.85818100	-0.51263600
C	-3.15496500	-4.08302900	-1.13468500
O	-2.06100400	-4.57657000	-1.41634000
C	-4.41749400	-4.82953400	-1.48463000
H	-4.93195000	-5.16883000	-0.59341700
H	-5.09229600	-4.22766900	-2.07932900
H	-4.11241800	-5.69298100	-2.05633500
C	-8.01343400	-1.99307900	0.74564600
H	-8.42810000	-2.06876100	-0.25421000
H	-8.14195000	-2.95367300	1.23343000
H	-8.58919400	-1.25810100	1.29362100
C	-3.41195500	6.09639700	0.78136700
C	-2.51309700	6.73047600	-0.06118900
C	-1.84994700	6.00569900	-1.04400500
C	-2.09940800	4.65237000	-1.19077400
C	-3.00496800	4.01567900	-0.34695400
C	-3.65443800	4.73536800	0.64143100
H	-3.91788000	6.65438000	1.54605800
H	-2.32170500	7.78073300	0.04924400
H	-1.14282500	6.49579500	-1.68825000
H	-4.34601100	4.24308200	1.30119100
C	-3.19267000	2.53497200	-0.56106700
H	-3.62404900	2.34141400	-1.53929600
H	-3.86524200	2.12243700	0.17238300
C	-1.40992800	3.77323000	-2.20113600
H	-2.11847200	3.39648700	-2.93310100
H	-0.63860500	4.31313800	-2.73748000

E= -3074.17875688 a.u.

The xyz coordinates for the lowest energy structure of **2''**

0 1

C	-4.31584600	2.11942300	-0.74758600
C	-3.27842100	3.11145100	-0.65257900
C	-2.24366100	3.14170500	-1.60390700
C	-2.26069700	2.17390400	-2.70448300
C	-3.30417800	1.22479100	-2.80133400
C	-4.33934900	1.19729300	-1.81766500
C	-4.65124100	1.67837500	0.59327500
C	-3.79723500	2.39961500	1.52618200
C	-2.94447600	3.28215200	0.75249800
C	-1.61801700	3.47401600	1.13320700

C	-0.88481600	3.38880300	-1.22328300
C	-0.91965000	1.78826900	-2.96630900
C	-0.61393300	0.45882300	-3.33695700
C	-1.68861700	-0.52475000	-3.44774400
C	-3.00177600	-0.14992900	-3.18394500
C	-3.86809400	-1.03198500	-2.41788700
C	-4.68626300	-0.19906700	-1.56317100
C	-4.99196900	-0.61726200	-0.26858700
C	-4.96674500	0.34030900	0.83125700
C	-3.29099100	1.75123600	2.65193700
C	-3.61406700	0.34933800	2.89350300
C	-4.44528400	-0.33768300	2.00153600
C	-4.13800900	-1.70802500	1.62909900
C	-4.48218500	-1.88037600	0.22509300
C	-3.69663000	-2.69028100	-0.59887600
C	-3.38117600	-2.25588600	-1.94741000
C	-2.00939500	-2.64378700	-2.22516100
C	-1.17948600	-1.79300900	-2.95673500
C	-0.05135500	2.51936900	-2.04515200
C	1.86171300	-1.64627500	-0.59244800
C	1.87842300	-0.12647400	-0.73077300
C	2.26453300	0.87270700	0.37521100
C	1.57840800	0.36016500	1.60943000
C	1.25155600	-0.97515200	1.82249800
C	0.19668800	-2.91891300	0.71783700
C	-0.15027000	-3.06357300	-0.65839900
C	0.70210500	-2.21712800	-1.43036200
C	0.19478000	-1.58460100	-2.54787100
C	0.51868200	-0.18414500	-2.78918500
C	1.31520100	0.51311000	-1.82374300
C	1.69563300	2.28723700	-0.18178000
C	0.62889100	2.93383100	0.69384800
C	0.28862300	2.41918400	1.97109200
C	0.78178000	1.19831500	2.46268900
C	-0.08345400	0.32569000	3.22821900
C	0.22973000	-1.03697300	2.84502200
C	-0.78465400	-1.99210800	2.79144800
C	-0.79872300	-2.95256200	1.67503100
C	-1.48482800	-3.30569600	-1.05162400
C	-2.51781400	-3.34326500	-0.05049400
C	-2.17948500	-3.16316000	1.29443000
C	-3.00727700	-2.33576900	2.15179000
C	-2.14182000	-1.62942700	3.08772200
C	-2.44625800	-0.30068700	3.44603000

C	-1.39223100	0.68278600	3.53438100
C	-1.91034800	1.95595300	3.03857300
C	-1.09459900	2.79982300	2.29410600
C	1.06080700	1.90436600	-1.52045900
C	-0.54771500	3.51569900	0.13908800
C	1.51854000	-2.14380200	0.89309500
C	3.74927100	1.13810800	0.54549300
C	4.70538600	0.31345800	1.12311500
C	4.10061700	2.40919500	0.06673000
C	6.03920100	0.71938200	1.17306300
H	4.41127500	-0.63647500	1.55735800
C	5.44299800	2.80880000	0.10491500
C	6.41787100	1.95987100	0.64975600
H	6.78537200	0.07212900	1.62563900
H	5.76304400	3.77127300	-0.26272600
N	2.95576000	3.11054800	-0.37077200
C	2.83088800	4.38789900	-0.90900600
O	1.73479000	4.79851900	-1.25866700
C	4.05274600	5.27556600	-1.05745900
H	4.54367100	5.46122000	-0.09692800
H	4.78796800	4.84672600	-1.74625000
H	3.69703500	6.22228400	-1.46558500
C	7.86652600	2.39133600	0.65903700
H	8.36411700	2.12508000	-0.28301400
H	7.96427200	3.47517400	0.78250100
H	8.42174600	1.90697400	1.46857300
C	2.61666600	-3.09501400	1.47388900
C	3.23431200	-2.22742300	-1.10689500
C	3.04210900	-4.14327400	0.47521700
C	3.34900900	-3.70603500	-0.82405500
C	3.74531500	-4.63190900	-1.79129800
C	3.85162400	-5.98665900	-1.46928800
C	3.54766200	-6.42128000	-0.17872300
C	3.13786500	-5.50055700	0.78806800
H	3.97308200	-4.29257000	-2.79918500
H	4.16324300	-6.70023300	-2.22709400
H	3.62085400	-7.47545300	0.07420800
H	2.89093800	-5.83868300	1.79184400
H	3.48197700	-2.48665000	1.76930900
H	2.23422000	-3.55336200	2.39168100
H	3.32224300	-2.02191400	-2.17840600
H	4.04759300	-1.68221700	-0.61186100

E= -3074.16621300 a.u.

The xyz coordinates for the lowest energy structure of **4.**

0 1

C	-3.78236200	1.68296800	-2.21239500
C	-2.57206400	1.55931300	-3.00155200
C	-2.18222200	0.31121200	-3.48754300
C	-2.98689500	-0.86640900	-3.20757100
C	-4.15000000	-0.74837400	-2.44312900
C	-4.55394300	0.55145700	-1.93490400
C	-3.53555000	2.67075200	-1.17123800
C	-2.17685400	3.15646100	-1.32767800
C	-1.58171400	2.46977800	-2.44734100
C	-0.23847100	2.07826500	-2.37022600
C	-0.78634800	-0.08186400	-3.42251800
C	-2.08505000	-1.98346900	-2.98324000
C	-2.37949600	-2.94132400	-2.01197200
C	-3.58850700	-2.82082100	-1.22125700
C	-4.45437900	-1.74359300	-1.42935400
C	-5.04583000	-1.05752700	-0.29375000
C	-5.10786400	0.35938100	-0.60571100
C	-4.86784600	1.30579800	0.39357900
C	-4.06803500	2.48241500	0.10518900
C	-1.39217400	3.41947900	-0.19947200
C	-1.94885000	3.22181800	1.12553200
C	-3.26166300	2.77264100	1.27986000
C	-3.57564300	1.78114400	2.29816000
C	-4.56237300	0.87287800	1.74714600
C	-4.50485200	-0.49213200	2.04775900
C	-4.74930900	-1.47541700	1.00569600
C	-3.84988700	-2.59456700	1.22212100
C	-3.28060900	-3.25698800	0.13345400
C	-0.72810900	-1.48654100	-3.11372400
C	0.26232600	-2.88378900	1.13688200
C	0.80743500	-2.71774900	-0.11547300
C	1.88587000	-1.66147500	-0.45999600
C	2.23336000	-0.63001500	0.73512500
C	1.25698200	-0.74849700	1.90030800
C	-0.70836200	-1.74965900	2.96187400
C	-1.69656600	-2.66279500	2.42009400
C	-1.10032100	-3.35378900	1.29150600
C	-1.88365600	-3.65030000	0.17104200
C	-1.32767500	-3.45208800	-1.14552300
C	-0.02116200	-2.96166800	-1.26611900
C	1.25311400	-1.04790200	-1.70254200
C	1.20834400	0.28103900	-1.99591500

C	1.77448400	1.41105400	-1.14024600
C	2.18818500	0.99693500	0.37061100
C	1.16582900	1.51437400	1.38860100
C	0.81727100	0.52663800	2.35717900
C	-0.32014000	0.65501300	3.16894300
C	-1.10842300	-0.51264200	3.47107600
C	-3.04402500	-2.29854000	2.39531000
C	-3.45626400	-1.00105500	2.90982000
C	-2.50652400	-0.12742400	3.44279900
C	-2.56741500	1.29333900	3.13121800
C	-1.20642200	1.77034000	2.97372100
C	-0.89672600	2.70660500	1.98333500
C	0.31284300	2.58243800	1.20179100
C	0.00423400	3.03979600	-0.16607900
C	0.58118100	2.39495900	-1.23267100
C	0.26969200	-1.94521700	-2.25372700
C	0.15727700	0.77413100	-2.85333200
C	0.50136900	-1.86655600	2.17860500
C	3.24276700	-2.26524800	-0.79826400
C	3.60247100	-3.03520900	-1.89580400
C	4.19858700	-1.88917000	0.14244500
C	4.93782600	-3.41589100	-2.05349000
H	2.85897700	-3.32730300	-2.63168200
C	5.54321800	-2.21819700	-0.04050200
C	5.91754300	-3.00513600	-1.14127700
H	5.22496600	-4.02002100	-2.90983400
H	6.30762800	-1.86559700	0.64151700
N	3.60076000	-1.11298500	1.16239700
C	4.04437200	-0.99498000	2.47700600
O	3.53712600	-0.19137900	3.24834900
C	5.15451700	-1.91150800	2.96590100
H	5.16438400	-2.88848000	2.47882600
H	6.13514100	-1.44324800	2.82619800
H	4.99921400	-2.03015100	4.03980400
C	7.36536000	-3.39767700	-1.32706900
H	8.03622100	-2.54577000	-1.16693500
H	7.66043500	-4.17922300	-0.61454600
H	7.54731800	-3.78517900	-2.33406300
C	3.57916600	1.63724100	0.72143000
C	2.99527100	2.05558400	-1.89155700
C	3.74121800	3.04786500	0.22392500
C	3.49504000	3.25737800	-1.14045600
C	3.66275700	4.52621200	-1.69537600
C	4.08877900	5.58746900	-0.89259200

C	4.33026700	5.38175800	0.46708400
C	4.15234400	4.11379900	1.02620400
H	3.45915900	4.68682200	-2.75177500
H	4.22308400	6.57469500	-1.32652500
H	4.65213300	6.20910800	1.09391500
H	4.33661000	3.95196000	2.08573100
H	4.35900500	1.01610100	0.26174900
H	3.72495100	1.57956600	1.80073400
H	3.78095600	1.29279900	-1.98750900
H	2.68316000	2.31669600	-2.90767900

E = -3074.23148240 a.u.

References

1. a) A. Fernandez-Ramos, J. A. Miller, S. J. Klippenstein and D. G. Truhlar, *Chem. Rev.*, 2006, **106**, 4518–4584; b) S.-H. Li, Z.-J. Li, W.-W. Yang and X. Gao, *J. Org. Chem.*, 2013, **78**, 7208–7215.

2. Y. Xiao, S.-E Zhu, D.-J. Liu, M. Suzuki, X. Lu and G.-W. Wang, *Angew. Chem. Int. Ed.*, 2014, **53**, 3006–3010.
